# Low Dose Soft X‐Ray Remotely Triggered Lanthanide Nanovaccine for Deep Tissue CO Gas Release and Activation of Systemic Anti‐Tumor Immunoresponse

**DOI:** 10.1002/advs.202004391

**Published:** 2021-04-08

**Authors:** Youbin Li, Mingyang Jiang, Zhiming Deng, Songjun Zeng, Jianhua Hao

**Affiliations:** ^1^ Synergetic Innovation Center for Quantum Effects and Application, Key Laboratory of Low‐dimensional Quantum Structures and Quantum Control of Ministry of Education, Key Laboratory for Matter Microstructure and Function of Hunan Province, School of Physics and Electronics Hunan Normal University Changsha 410081 P. R. China; ^2^ Department of Applied Physics The Hong Kong Polytechnic University Hong Kong 999077 P. R. China

**Keywords:** activated systemic anti‐tumor immunotherapy, elicited tumor oxidative stress, gas therapy, lanthanide scintillator‐based nanovaccine, reversed immunosuppressive tumor microenvironment, soft X‐ray‐activated CO release

## Abstract

Gas‐based therapy has emerged as a new green therapy strategy for anti‐tumor treatment. However, the therapeutic efficacy is still restricted by the deep tissue controlled release, poor lymphocytic infiltration, and inherent immunosuppressive tumor microenvironment (TME). Herein, a new type of nanovaccine is designed by integrating low dose soft X‐ray‐triggered CO releasing lanthanide scintillator nanoparticles (ScNPs: NaLuF_4_:Gd,Tb@NaLuF_4_) with photo‐responsive CO releasing moiety (PhotoCORM) for synergistic CO gas/immuno‐therapy of tumors. The designed nanovaccine presents significantly boosted radioluminescence and enables deep tissue CO generation at unprecedented tissue depths of 5 cm under soft X‐ray irradiation. Intriguingly, CO as a superior immunogenic cell death (ICD) inducer further reverses the deep tissue immunosuppressive TME and concurrently activates adaptive anti‐tumor immunity through efficient reactive oxygen species (ROS) generation. More importantly, the designed nanovaccine presents efficient growth inhibition of both local and distant tumors via a soft X‐ray activated systemic anti‐tumor immunoresponse. This work provides a new strategy of designing anti‐tumor nanovaccines for synergistic deep tissue gas‐therapy and remote soft X‐ray photoactivation of the immune response.

## Graphical abstract

A novel soft X‐ray remotely stimulated lanthanide scintillator‐based CO releasing system is designed. The nanovaccine presents efficient CO generation ability, which can further realize deep tissue gas therapy, activate a systemic anti‐tumor immune‐response, and reverse an immunosuppressive tumor microenvironment. The findings demonstrate a new strategy of designing anti‐tumor nanovaccines for soft X‐ray triggered deep tissue gas therapy and immune response.

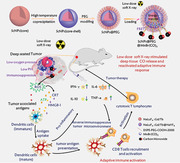

## 1 Introduction

Anti‐tumor immunotherapies have received large scientific breakthrough due to their manageable side effect in recent years.^[^ ^1^ ^]^ Via affecting autoimmune systems, immunotherapy has shown promising effect on eliminating the malignant cells.^[^ ^2^ ^]^ Nevertheless, owing to low pH, hypoxic stress features of deep and inner parts of solid tumors, the existing immunotherapy strategy is still limited by the immunosuppressive tumor microenvironment (TME) in the solid tumor, lacking tumor immune surveillance and resulting in immune escape and low response rates.^[^ ^3^, ^4^, ^5^, ^6^ ^]^

Recent studies indicated that some nanomaterials with light‐stimulated anti‐tumor therapy strategies could trigger ICD and concomitantly induce anti‐tumor immune response through reactive oxygen species (ROS) activation, thus enhancing anti‐tumor immunotherapy.^[^ ^7^, ^8^, ^9^, ^10^, ^11^, ^12^, ^13^, ^14^, ^15^, ^16^ ^]^ For example, Liu and co‐workers presented an artificial Cu_2−_ *_x_*Te nanoenzyme as an immunostimulant to enhance immunotherapy of cancer using the 1064 nm second near‐infrared (NIR‐II) light irradiation.^[^ ^7^ ^]^ Wang et al. demonstrated an obvious therapeutic effect by a photothermal triggered Au nanoprobe with immunological response via utilizing 1064 nm NIR‐II light.^[^ ^9^ ^]^ Liu et al. reported the NIR light (980 nm) responsive AIEgen‐coupled upconversion (NaYF_4_:Yb/Tm@NaYF_4_) nanoparticles (UCNPs) as an immunostimulant for anti‐tumor immunity through dual model ROS activation.^[^ ^16^ ^]^ However, owing to the inherent large photon scattering losses of NIR light below 1100 nm in biotissue,^[^ ^17^, ^18^ ^]^ the immunotherapy efficiency is still limited by the restricted penetration depth (<1 cm) for deep‐seated tumor and the immunosuppressive TME.^[^ ^9^ ^]^ Therefore, developing a new type of nanovaccine to activate the anti‐tumor immunity response in deep seated solid tumor and reverse the deep tissue immunosuppressive TME is urgently demanded for cancer immunotherapy.

On the other hand, among the various gas‐based cancer therapy systems,^[^ ^19^ ^]^ CO gas sensitized anti‐tumor therapy as a new green treatment strategy, presents a positive therapy effect on cancer cells while protecting normal cells from apoptosis,^[^ ^20^, ^21^ ^]^ opening up new frontiers in cancer therapy owing to its unique therapeutic efficacy.^[^ ^22^, ^23^, ^24^ ^]^ However, the therapy efficiency is also restricted by the immunosuppression TME of tumor and deep tissue controllable gas release efficiency. Therefore, in order to achieve effective deep tissue controlled CO release, remote light‐stimulated nanosystems with non‐invasive and controllable exogenous stimulus properties, were developed as attractive stimuli responsive nanoplatform for on‐demand generation of CO gas.^[^ ^25^, ^26^, ^27^, ^28^, ^29^, ^30^ ^]^ Although some progresses have been achieved, the traditional PhotoCORM systems are usually triggered by ultraviolet (UV) and visible light, unavoidably resulting in shallow tissue penetration and impeding their bioapplications.^[^ ^25^, ^26^ ^]^ To solve this problem, the NIR light‐responsive PhotoCORM^[^ ^24^, ^27^ ^]^ nanoplatforms were developed for deep tissue CO generation. For example, Chen and co‐workers have designed an 808 nm NIR light‐responsive MnCO‐graphene oxide nanoplatform for on‐demand releasing of CO in cell.^[^ ^24^ ^]^ And, a lanthanide‐based NaGdF_4_:Tm,Yb@NaGdF_4_‐PhotoCORM nanoplatform was also developed for CO release under NIR laser (980 nm) excitation.^[^ ^27^ ^]^ However, the limited tissue penetration depth (<1 cm) of NIR light is still a hurdle restricting for deep tissue CO release in tumor therapy.^[^ ^17^, ^18^ ^]^ Therefore, a new controllable strategy for realizing deep tissue CO generation is highly desirable for deep‐seated solid tumor therapy. Moreover, previous study demonstrated that the CO gas can enhance the chemosensitivity of cancer cells by elevating oxidative stress and promoting ROS generation.^[^ ^20^ ^]^ Therefore, it is expected that the CO gas therapy can introduce a tremendous infusion of hope as a new ICD inducer to reverse the immunosuppression TME and activate the adaptive anti‐tumor immunoresponse, which has not yet been explored.

To this end, seeking a new photo‐stimulated nanovaccine for simultaneously realizing deep tissue controllable generation of CO, activating the systemic anti‐tumor immune response, and reversing the immunosuppression TME of deep‐seated solid tumor is significantly important, which still remains great challenging. It is noteworthy that high energy X‐ray, as one kind of ionized radiation source capable of overcoming penetration depth limitation in tissues, has achieved significant progresses to reach and destroy deep‐seated tumors.^[^ ^31^, ^32^, ^33^ ^]^ To solve the penetration depth issues suffered by both gas and anti‐tumor immunity therapy, we have proposed a soft X‐ray stimulated strategy to achieve deep tissue on‐demand generation of CO and reverse the immunosuppression TME.

**Scheme 1:**
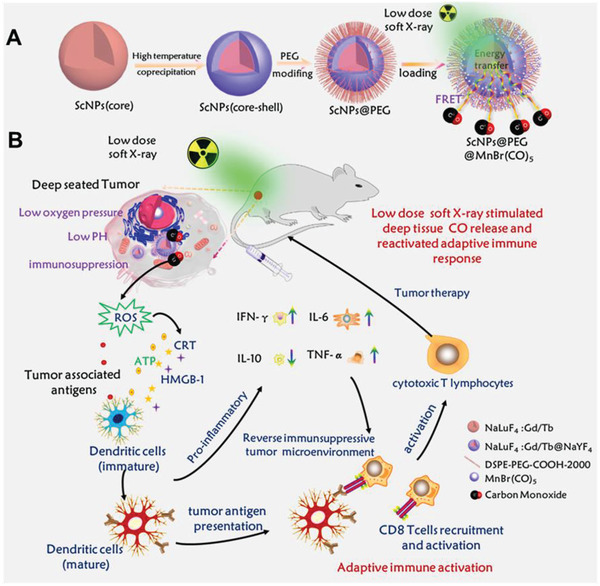
A) Schematic design of lanthanide‐based ScNPs‐PhotoCORM nanovaccine. B) Soft X‐ray stimulated deep tissue CO release for in vivo gas‐sensitized and activated immunotherapy of cancer with reversed immunosuppressive TME.

Herein, a new type of soft X‐ray stimulated nanovaccine through caging the MnCO PhotoCORM with lanthanide‐based ScNPs for deep tissue CO gas therapy and activation of the anti‐tumor immunity (**Scheme** **1**) was developed. Around 7.5‐fold improvement of the X‐ray excited radio‐fluorescence (XEF) intensity was realized by adjusting the Tb^3+^ doping concentration and core‐shell strategy. Upon soft X‐ray irradiation, on‐demand deep tissue (≈5 cm) release of CO was successfully achieved. More importantly, the efficient CO release induced intracellular ROS generation was achieved and further reversed the immunosuppressive TME, subsequently boosting the systemic anti‐tumor immune response to prevent both primary tumor and distant tumor growth, which has not yet been achieved in gas therapy related systems. This work provides a new soft X‐ray light stimulated strategy for synergistically integrating gas‐immunological response and anti‐tumor immunity into a single nanovaccine for effective anti‐tumor therapy at unprecedented tissue depths.

## 2 Results and Discussion

### 2.1 Design of Lanthanide Scintillator‐Based Nanovaccine

**Figure 1:**
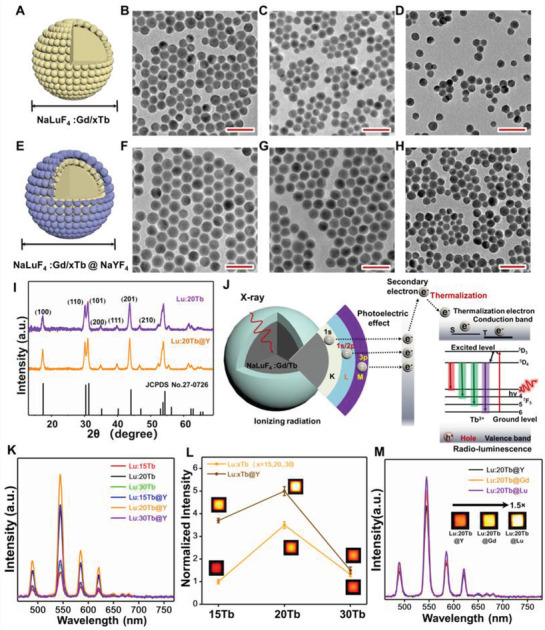
Structure and XEF properties of lanthanide‐based ScNPs. A–D) Scheme and TEM images of NaLuF_4_:Gd,xTb (*x* = 15, 20, 30) core nanoparticles, respectively. All the scale bars are 50 nm. E–H) Scheme and TEM images of NaLuF_4_:Gd,xTb@NaYF_4_ (*x* = 15, 20, 30) core‐shell nanoparticles, respectively. All the scale bars are 50 nm. I) XRD patterns of the as‐prepared NaLuF_4_:Gd,20Tb core and NaLuF_4_:Gd,20Tb@NaYF_4_ core‐shell nanoparticles. J) Schematic illustration of the XEF mechanism in NaLuF_4_:Gd,Tb@NaYF_4_ core‐shell nanoparticles. K) The XEF spectra of the NaLuF_4_:Gd,xTb (*x* = 15, 20, 30) core and NaLuF_4_:Gd,xTb@NaYF_4_ core‐shell nanoparticles under X‐ray irradiation, X‐ray voltage tube: 50 kVp. L) Corresponding XEF intensity of the synthesized core and core‐shell nanoparticles in Figure 1K. The insets show the in vitro phantom X‐ray luminescence images of the NaLuF_4_: Gd,xTb core, and NaLuF_4_: Gd,xTb@NaYF_4_ core‐shell nanoparticles. M) The XEF spectra of NaLuF_4_: Gd,20Tb@NaLnF_4_ (Ln = Lu, Gd, Y) core‐shell nanoparticles. The insets show the corresponding in vitro phantom X‐ray luminescence images.

The lanthanide scintillator‐based nanovaccine (Scheme 1) was designed by integrating the core‐shell (NaLuF_4_:Gd,xTb@NaYF_4_) ScNPs with MnBr(CO)_5_ PhotoCORM for soft‐ray triggered deep tissue on‐demand CO release, adaptive immune activation, and reversed immunosuppressive TME. Initially, lanthanide‐based core (NaLuF_4_:Gd,xTb, *x* = 15, 20, 30) and core‐shell (NaLuF_4_:Gd,xTb@NaYF_4_) ScNPs with various Tb^3+^ doping concentrations were prepared by using a high temperature co‐precipitation method. Transmission electron microscopy (TEM) and X‐ray powder diffraction (XRD) analysis of the core and core‐shell nanoparticles were performed to evaluate the particle size and crystal structure. As shown in **Figure** **1A**,B, TEM image of the as‐synthesized NaLuF_4_: Gd,15Tb core nanoparticles presented the extremely uniform spherical structure with an average size of ~18 nm. High resolution (HR) TEM result (Figure S1A, Supporting Information) of the core only ScNPs presented high crystallinity, and the crystal lattice distant was measured to be ≈0.31 nm, matching well with the (111) crystal face of the hexagonal phase NaLuF_4_.^[^ ^34^ ^]^ For comparison, various Tb^3+^ doped core nanoparticles were prepared (Figure 1C,D and Figure S1B,C, Supporting Information), proving similar crystal phase structure. Then, a core‐shell strategy was adopted to improve the XEF efficiency of the ScNPs by reducing the surface quenching effect. The TEM (Figure 1E–H) and HRTEM (Figure S1D–F, Supporting Information) results of the core‐shell nanoparticles both showed similar crystal lattice fringes and increased particle size from ≈18 to ≈21 nm, revealing the formation of core‐shell ScNPs nanoparticles with hexagonal phase structure. Energy dispersive X‐ray spectroscopy (EDS, Figure S1G, Supporting Information) of NaLuF_4_:Gd,20Tb@NaYF_4_ core‐shell nanoparticles further revealed the presence of Na, Lu, F, Gd, Tb, and Y elements. XRD measurements of the core and core‐shell nanoparticles (Figure 1I and Figure S2A, Supporting Information) demonstrated that both the core and core‐shell ScNPs displayed the pure hexagonal phase structure (JCPDS: 27–0726) with 15% Tb doping concentration. By increasing the Tb^3+^ doping concentration, no distinct phase change was observed, further proving the formation of pure hexagonal phase structure. And, with increased Tb doping contents (Figure S2B, Supporting Information), the prominent diffraction peak of the (211) facet was shifted to a lower diffraction angle, which was ascribed to the substitution of Lu^3+^ (*r* = 1.159 Å)^[^ ^35^ ^]^ by larger Tb^3+^ (1.283 Å).^[^ ^35^ ^]^ In addition, NaLuF_4_: Gd,20Tb core nanoparticles coated by various shells (NaGdF_4_ and NaLuF_4_) were also prepared. As shown in Figure S3, Supporting Information, all of the samples presented the similar structure with NaLuF_4_@NaYF_4_.

As is known, the scintillator is commonly employed as an energy transducer to convert the high energy photons (e.g., X‐ray light) into UV/visible light. As demonstrated in Figure 1J, the general XEF mechanism of X‐ray‐activated lanthanide‐based ScNPs can be mainly divided into three consecutive steps: conversion, transport, and luminescence.^[^ ^36^ ^]^ Initially, large numbers of free electrons and holes are created from the inner shell of the heavy atoms under soft X‐ray irradiation. This is mainly attributed to the X‐ray induced photoelectric effect and Compton scattering.^[^ ^36^ ^]^ Subsequently, the mass of hot electrons and holes are immediately thermalized and migrated to the conduction and valance band, respectively.^[^ ^36^ ^]^ Finally, XEF is generated by radiative combination of trapped electron‐hole pairs. Then, to reveal the XEF, the luminescence spectra of the as‐prepared core and core‐shell ScNPs were studied under X‐ray irradiation. As shown in Figure 1K, the XEF spectra of the ScNPs showed four typical emission peaks located at 489, 546, 584, and 620 nm, which were originated from electron transitions of ^5^D_4_ to ^7^F_J_ (*J* = 6, 5, 4, 3) of Tb^3+^ ions, respectively. The XEF emission intensity of the core‐shell nanoparticles was improved by ≈5 times by increasing the doping concentration of Tb^3+^ from 15% to 20% mol (Figure 1L). And the emission intensity was tended to decrease at higher Tb^3+^ doping concentration, owing to the concentration quenching effect and potential decreased absorption efficiency of Lu elements substituted by Tb (Tb_L‐edge_: 8.679 keV; Lu_L‐edge_: 10.87 keV).^[^^]^ Because the XEF efficiency is mainly dependent on the X‐ray attenuation coefficients, which is highly related to the high atomic number (Z) of elements. To further reveal this, the XEF properties of the core‐shell ScNPs coated with different inert shells were evaluated. As shown in Figure 1M, the XEF spectra and in vitro phantom X‐ray luminescence images of the NaLuF_4_:Gd,20Tb@NaLuF_4_ nanoparticle (Lu_L‐edge_: 10.87 keV) presented higher XEF efficiency than the NaLuF_4_:Gd,20Tb@NaGdF_4_ (Gd_L‐edge_: 8.393 keV) and NaLuF_4_:Gd,20Tb@NaYF_4_ (Y_L‐edge_: 2.369 keV) core‐shell nanoparticles.^[^^]^ And a significant enhancement (≈1.5‐fold) in XEF intensity was achieved, further revealing the high Z of lanthanide element dependent XEF improvement.

### 2.2 Soft X‐Ray Stimulated Deep Tissue CO Release

**Figure 2:**
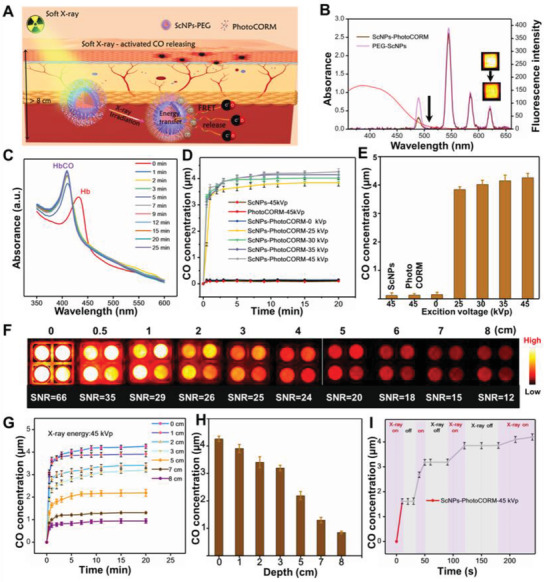
Soft X‐ray stimulated CO release. A) Schematic illustration of the deep tissue CO generation upon soft X‐ray light irradiation. B) The XEF spectra of the PEG‐modified NaLuF_4_:Gd,20Tb@NaLuF_4_ core‐shell nanoparticles, PhotoCORM loaded PEG‐NaLuF_4_:Gd,Tb@NaLuF_4_ core‐shell nanoparticles, and the absorbance spectra of the prepared PhotoCORM. C) The UV–vis absorption monitoring of CO release process from the ScNPs‐PhotoCORM nanovaccine in PBS under soft X‐ray light irradiation. D) CO release curves of the ScNPs, and PhotoCORM under 45 kVp X‐ray light irradiation and ScNPs‐PhotoCORM nanovaccine under X‐ray irradiation with different tube voltages. E) The quantitative X‐ray triggered CO releasing concentration in (D). F) In vitro phantom optical images of ScNPs‐PhotoCORM nanovaccine covered with different thicknesses of pork slices under soft X‐ray irradiation and the corresponding SNR values. G) Soft X‐ray triggered CO release profiles of the ScNPs‐PhotoCORM nanovaccine covered with different thicknesses of pork tissues. H) The corresponding quantitative release content of CO triggered by soft X‐ray in (G). I) The on‐demand controllable CO releasing behavior triggered by switching on/off X‐ray irradiation (Tube voltage: 45 kVp).

The NaLuF_4_:Gd,20Tb@NaLuF_4_ ScNPs with high performance XEF were first converted into aqueous phase with DSPE‐PEG‐COOH‐2000 surface modification for biomedical application. The XEF intensity of the PEG‐modified ScNPs and in vitro fluorescence images (Figure S4, Supporting Information) demonstrated a slight luminescence intensity decrease, owing to the water‐induced fluorescence quenching effect. Then, the photostability (Figure S5, Supporting Information) of the PEG‐modified ScNPs in phosphate buffered saline (PBS), water, and fetal bovine serum under continuous soft X‐ray irradiation for 2 h was investigated, revealing the high photostability of XEF with low photobleaching degree of 1.2%. Subsequently, the PhotoCORM was incorporated into the PEG‐ScNPs to form the ScNPs‐PhotoCORM nanovaccine. As shown in **Figure** **2A**,B, the UV–vis absorption spectra of the PhotoCORM were well overlapped with the XEF spectrum of the PEG‐ScNPs. When compared to the PEG‐ScNPs, the emission peaks at 475–505 nm of ScNPs‐PhotoCORMs showed an obvious decrease, and the green/red emissions remained unchanged, indicating the efficient fluorescence resonance energy transfer (FRET, 60%) of the blue light (475–505 nm) from ScNPs to PhotoCORM to generate CO under soft X‐ray irradiation. To further investigate the ability of soft X‐ray light‐triggered CO release, different concentrations of ScNPs were used for soft X‐ray induced in vitro phantom optical imaging. As shown in Figure S6, Supporting Information, the XEF signal was gradually enhanced with increasing the ScNPs‐PhotoCORM concentrations and elevating the X‐ray tube voltage from 25 to 45 kVp. The time‐dependent XEF properties of ScNPs‐PhotoCORM were further studied with X‐ray irradiation at different irradiation times and 45 kVp. Bright XEF was achieved with long time irradiation (Figure S6, Supporting Information).

Then, soft X‐ray light responsive CO releasing behavior was first evaluated by modifying X‐ray tube voltage. The CO release concentration was calculated by using the hemoglobin (Hb) method according to Beer–Lambert law.^[^ ^24^ ^]^ The characteristic absorption band of Hb at 430 nm was disappeared accompanied by the generation of the characteristic absorption band of carboxyhemoglobin (HbCO) at 410 nm, suggesting the generation of CO (Figure 2C). As shown in Figure 2D,E, the ScNPs and PhotoCORM solutions presented no CO generation under X‐ray irradiation for 20 min. In contrast, ScNPs‐PhotoCORM was highly responsive to X‐ray light to generate CO. With elevating the X‐ray tube voltage from 25 to 45 kVp, the CO release concentration was increased from 3.6 to 4.4 µm, which could be attributed to the improved XEF intensity and efficient FRET from ScNPs to PhotoCORM. The depth‐dependent CO releasing properties of ScNPs‐PhotoCORM under soft X‐ray irradiation at 45 kVp were investigated by covering various thicknesses of pork slices. As shown in Figure 2F, Figures S7 and S8, Supporting Information, a high signal‐to‐noise ratio (SNR, 66) was observed without covering pork. Bright XEF and high SNR of 12 were still achieved with increasing the pork thickness even up to 8 cm, revealing the profound penetration nature of X‐ray light. And the CO release concentration (Figure 2G,H) can even reach ≈3 µm at 3 cm depth, which is unattained by the traditional NIR or NIR‐II light stimulated gas releasing systems. More importantly, the ScNPs‐PhotoCORM also showed strong CO generation ability even at an unprecedented tissue depth of 8 cm, and the level of the generated CO reached nearly ≈1 µm under soft X‐ray irradiation for 60 s.

As is known, the controlled release of CO is also significant for efficient CO sensitized anti‐tumor therapy as well as reducing the CO poisoning risk. As illustrated in Figure 2I, when X‐ray light was switched on, a burst CO release was observed after 30 s of continuous irradiation. In contrast, the CO release rate was completely stopped when the X‐ray light was switched off, suggesting the on‐demand controlled CO release nature via intermittent X‐ray irradiation. Notably, the traditional high dose X‐ray (>5 Gy) irradiation was usually required to achieve desirable treatment effects in clinic radiotherapy.^[^ ^38^ ^]^ Thus, for X‐ray triggered nanomedicine, the low dose is highly required for clinical application to reduce the radiation risk induced by the traditional high dose X‐ray. Fortunately, in our designed soft X‐ray triggered ScNPs‐PhotoCORM system, CO generation was only realized under soft X‐ray irradiation with dose rate of 490 μGy s^−1^ for 1–5 min, which is significantly lower than the dose (>5 Gy) used in clinic radiation therapy.^[^ ^38^ ^]^ These results demonstrate that the soft X‐ray light triggered ScNPs‐PhotoCORM system with controllable CO release and significant breakthrough in depth limitation is of vital importance for deep tissue CO gas mediated anti‐tumor therapy.

### 2.3 In Vitro Soft X‐Ray Activated CO Gas Therapy and Gas‐Mediated ROS Generation and ICD

Prior to evaluating the X‐ray induced CO therapeutic performance in vivo, we first estimated the cytotoxicity and feasibility application in vitro. As shown in Figure S9A, Supporting Information, the 4T1 cells maintain high cell viability (>95%) after incubation with ScNPs‐PhotoCORM (0–1 mg mL^−1^) nanovaccine for 24 h, revealing the high biocompatibility and low cytotoxicity of ScNPs‐PhotoCORM nanovaccine in vitro. Afterward, in vitro CO mediated gas therapy was performed. As shown in Figure S9B, Supporting Information, the cell viability of the control groups and the X‐ray treated ScNPs‐PhotoCORM nanovaccine with different times of X‐ray irradiation was performed. The survival rate decreased through elevating the irradiation time, which was mainly attributed to the enhanced CO therapy effect. Besides, as shown in Figure S9C, Supporting Information, the survival rate of the 4T1 cells treated with ScNPs‐PhotoCORM plus X‐ray irradiation was dramatically decreased when compared with the other groups (#1. Control; #2. X‐ray irradiation; #3. ScNPs‐PhotoCORM; #4. X‐ray and ScNPs). Then, we demonstrated the live/dead cell double‐staining test (Figure S9D, Supporting Information) by using Calcein‐AM and propidium iodide (PI), further verifying the efficient in vitro gas‐sensitized cancer cell death.

**Figure 3:**
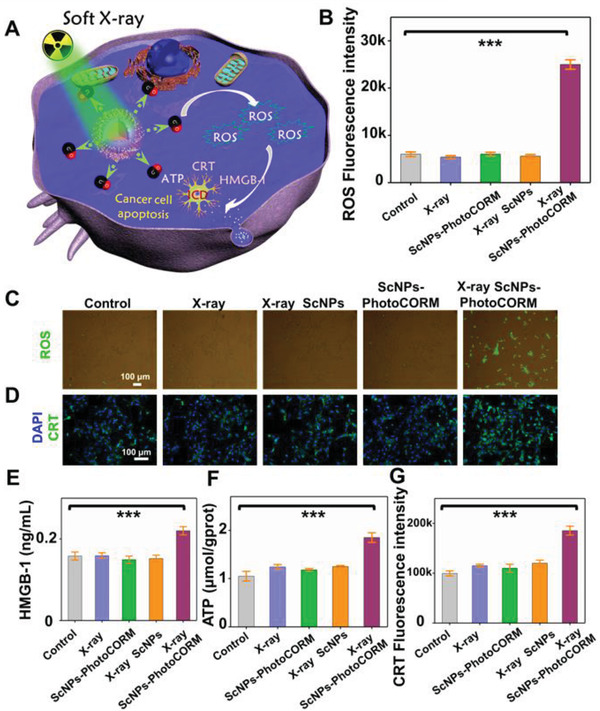
In vitro CO gas therapy induced ROS generation and ICD. A) Schematic illustration of CO gas therapy induced ICD under soft X‐ray irradiation. B) Statistical data of mean intracellular fluorescence intensity of DCFH‐DA in (C). C) Intracellular ROS fluorescence image after different treatment by using the DCFH‐DA assay. D) The expression of CRT in 4T1 cells imaged after different treatments through immunofluorescence staining. E) The release levels of the HMGB‐1 from cells after different treatments. F) The release amount of ATP from cells after different treatments. G) Statistical data of CRT‐positive fluorescence intensity acquired from (D). (^***^ *P* < 0.001).

It is noted that the dying tumor cells may release tumor associated antigens (TAA) and damage‐associated molecular patterns (DAMPs) during the process of ICD.^[^ ^39^, ^40^, ^41^ ^]^ And, the TAA can elicit the antigen‐specific T cell response, while DAMPs can act as inducer to release “eat” me signal to promote the engulfment of tumor antigens by dendritic cells (DCs) and enable the maturation of DCs.^[^ ^39^, ^40^, ^41^ ^]^ Then, the T cells can be activated and initiate adaptive anti‐tumor immune response. Recently, CO mediated gas therapy was found to promote cancer cell apoptosis through activating the mitochondrial ROS signaling pathway.^[^ ^21^ ^]^ It is noted that the excessive ROS can further induce ICD. And during ICD, the release of DAMPs, including adenosine triphosphate (ATP, a chemoattractant for antigen‐presenting cells^[^ ^7^, ^42^ ^]^), calreticulin (CRT), and high mobility group box 1 (HMGB‐1) occurred, further resulting in the activation of adaptive anti‐tumor immune response.^[^ ^7^ ^]^ Therefore, it is expected that the developed soft X‐ray stimulated nanoplatform, which breaks through the depth barrier of the conventional NIR light, can be used as promising nanovaccine for deep tissue immunity activation and reversing the immunosuppressive TME (**Figure** **3A**).

To reveal the CO‐induced immune response, CO gas therapy triggered ROS generation was first evaluated by using the 2,7‐dichlorodi‐hydrofluorescein diacetate (DCFH‐DA) assay. As shown in Figure 3B,C, an efficient ROS generation (≈5 fold) was observed in the group treated with ScNPs‐PhotoCORM nanovaccine plus X‐ray irradiation compared with those of other groups, indicating the efficient ROS generation capability stimulated by CO gas. Later, the release behavior of DAMPs is further assessed to validate whether CO gas therapy can cause ICD. As shown in Figure 3D,G, the efficient surface‐exposed CRT in cancer cells was observed when treated with ScNPs‐PhotoCORM nanovaccine plus X‐ray irradiation, which was much higher than other groups without CO generation. Besides, the significant release of HMGB‐1 and ATP was also observed from ScNPs‐PhotoCORM plus X‐ray irradiation group (Figure 3E,F). These findings further indicate that the soft X‐ray induced CO gas therapy can effectively trigger ICD and release DAMPs from 4T1 cancer cells, which may offer potential antigenic stimulation for deep tissue immune system activation.

### 2.4 Soft X‐Ray Triggered Gas‐Sensitized Anti‐Tumor Therapy

**Figure 4:**
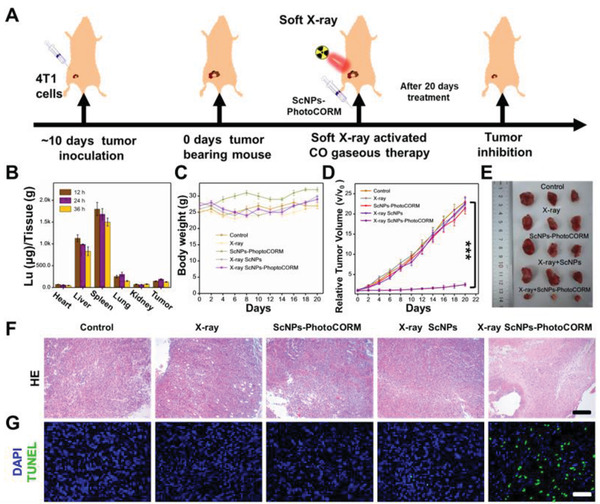
Soft X‐ray stimulated CO generation for gas‐sensitized anti‐tumor therapy in vivo. A) Schematic diagram of soft X‐ray‐activated CO gas therapy of tumor in vivo. B) Quantitative biodistribution of Lu in 4T1 tumor‐bearing mice after 12, 24, and 36 h intravenous injection of ScNPs‐PhotoCORM nanovaccine. The data was measured by ICP‐MS. C) The trend of body weight changes in different groups (Control; X‐ray irradiation; ScNPs‐PhotoCORM; X‐ray plus ScNPs; X‐ray plus ScNPs‐PhotoCORM) of 4T1 tumor‐bearing mice. D) The tumor growth curves of the 4T1 tumor‐bearing mice after different treatments in 20 days, ^***^ *P* < 0.001. E) Digital pictures of tumors removed from the 4T1 tumor‐bearing mice after various treatments for 20 days. F,G) HE and TUNEL stained images of tumor slices collected from different test groups, respectively. Scale bar: 50 µm.

Benefiting from the excellent in vitro therapy effect, in vivo CO gas sensitized anti‐tumor therapy was further demonstrated (**Figure** **4A**). Owing to the high biocompatibility of the nanovaccine, in vivo optical‐guided bioimaging was first demonstrated after subcutaneous injection of the nanovaccine in the tumor site. As shown in Figure S10, Supporting Information, the fluorescence signal intensities were gradually enhanced with improving tube voltage and exposure time, revealing the excellent optical properties of the nanovaccine for potential CO generation in vivo. Before in vivo application, the quantitative time‐dependent biodistribution of the ScNPs‐PhotoCORM nanovaccine in vivo was then investigated. The tumor‐bearing mice were dissected to gain the main organs (liver, spleen, kidney, heart, and lung) and tumors after intravenous injection of the nanovaccine at different time points for inductively coupled plasma mass spectrometry (ICP‐MS) analysis. As shown in Figure 4B, the ScNPs‐PhotoCORM nanovaccine was mainly accumulated in the liver, spleen, lung organs. And large amounts of nanovaccines in the tumor site were also observed, indicating the efficient accumulation of the nanovaccine in the tumor. The soft X‐ray induced CO gas therapy of cancer in vivo was then evaluated. The 4T1 tumor‐bearing mice were first divided into five groups in random and treated with PBS, X‐ray, ScNPs‐PhotoCORM, X‐ray plus ScNPs, X‐ray plus ScNPs‐PhotoCORM. All groups present no apparent losses of weight after 20 days of treatment (Figure 4C). Moreover, the tumor growth rates of the X‐ray, ScNPs‐PhotoCORM, X‐ray plus ScNPs treated groups were similar to the control group (Figure 4D,E), indicating the lack of therapeutic effect without CO generation. In contrast, the ScNPs‐PhotoCORM nanovaccine plus X‐ray irradiation group showed significant tumor inhibition efficiency, indicating the efficient CO mediated gas therapy of tumor in vivo. Subsequently, the therapeutic efficiency was further confirmed by hematoxylin and eosin (HE) and transferase dUTP nick end labeling (TUNEL) staining assay. As shown in Figure 4F,G, obvious cell apoptosis was observed in the ScNPs‐PhotoCORM plus X‐ray group, and limited cell apoptosis was found in those of other groups, revealing the effective soft X‐ray triggered CO gas‐therapy of tumor.

Additionally, the traditional 980 nm laser triggered UCNPs‐PhotoCORM nanoplatform for CO release was developed to reveal the advantages of the soft X‐ray induced deep tissue CO gas therapy in vitro and in vivo. As demonstrated in Figure S11, Supporting Information, both in vitro and in vivo therapeutic results of the soft X‐ray activated CO gas therapy show significant inhibition effect on tumor growth at ≈5 cm tissue depth, which is higher than the commonly used 980 nm laser mediated CO gas therapy (≈1 cm), proving the benefit of the deep tissue CO gas therapy under soft X‐ray irradiation.

For in vivo CO content measurement, the tumor tissues of 4T1 tumor‐bearing mice after various treatments were quickly collected for CO detection by using the endogenous CO assay kit. As shown in Figure S12, Supporting Information, the 4T1 tumor‐bearing mice with PBS, X‐ray, ScNPs‐PhotoCORM, X‐ray plus ScNPs treatment showed limited CO release content in the tumor site. In contrast, large amount of CO contents were detected in the X‐ray plus ScNPs‐PhotoCORM nanovaccine group, proving the efficient CO generation ability in vivo. It should be noted that CO possesses higher (≈200–300 times) affinity than oxygen to bind Hb in red blood cells.^[^ ^43^ ^]^ Thus, the oxygen carrying capacity of Hb is impaired upon small amount CO exposure, resulting in tumor oxygen deprivation.^[^ ^43^ ^]^ To validate this, the expression levels of hypoxia inducible factor (HIF‐1*α*) and HbCO content in the tumor site were measured. As shown in Figure S13, Supporting Information, both the HbCO and HIF‐1*α* levels were gradually increased with increasing therapy time, further revealing the efficient CO release in the tumor site.

We have then demonstrated CO induced ICD in vivo, the expression levels of HMGB1 and CRT in the tumor site were further demonstrated. As shown in Figures S14 and S15, Supporting Information, 4T1 tumor‐bearing mice in group treated with ScNPs‐PhotoCORM nanovaccine plus X‐ray showed significantly enhanced expression of CRT than the other groups. Besides, after soft X‐ray activated CO gas therapy, the HMGB1 was gradually diffused from the cell nucleus to the cytoplasm. Taken together, these results further demonstrated that the soft X‐ray induced ROS can also induce ICD in vivo.

**Figure 5:**
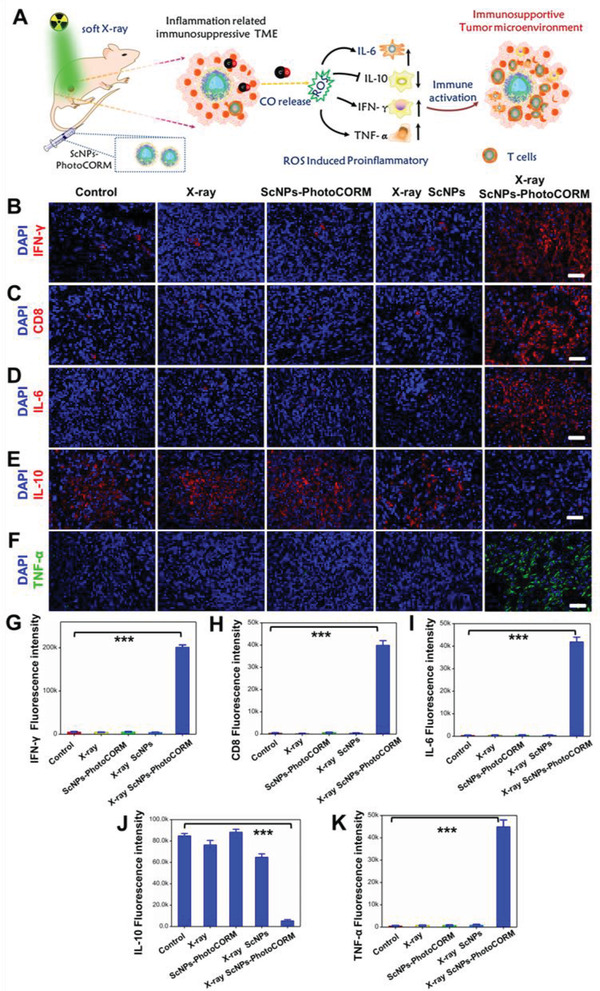
In vivo CO gas therapy activated deep tissue anti‐tumor immunoresponse and reversed immunosuppressive TME. A) Schematic illustration of CO gas induced immunity activation and the corresponding immune‐modulating mechanism. Immunofluorescence images of tumor slices obtained from tumor‐bearing mice treated with different groups. Tumor slices were stained with B) IFN‐*γ*, C) CD8, D) IL‐6, E) IL‐10, and F) TNF‐*α* anti‐body, respectively. Scale bar: 50 µm. G–K) Statistical data of the IFN‐*γ*, CD8, IL‐6, IL‐10, and TNF‐*α* positive fluorescence intensity acquired from (B, C, D, E, and F), respectively. ^***^ *P* < 0.001.

It is well known that ICD is a key factor to initiate the adaptive anti‐tumor immunity.^[^ ^16^ ^]^ The released DAMPs during ICD serve as the important signals to promote DCs maturation.^[^ ^40^, ^41^, ^42^ ^]^ Whereafter, the maturation DCs can assist the TAAs to promote T cells to enhance lymphocytic infiltration and activate adaptive anti‐tumor immunity,^[^ ^40^, ^41^, ^42^ ^]^ further eliciting the secretion of interferon‐*γ* (IFN‐*γ*, an important role in the activation of T‐cell‐based anti‐tumor immunity) and CD8 (**Figure** **5A**). Encouraged by the results of in vitro and in vivo soft X‐ray induced ICD, we speculated that our X‐ray induced CO gas therapy can also act as an artificial nanovaccine to initiate the deep tissue adaptive anti‐tumor immunity. Thus, the tumor slices after different treatments were stained with IFN‐*γ* and CD8 anti‐body to investigate the activation of anti‐tumor immune response. As shown in Figure 5B,C, it was found that the control, X‐ray, ScNPs‐PhotoCORM, X‐ray plus ScNPs groups showed no expression of IFN‐*γ* and CD8. Significantly, a remarkable enhancement of IFN‐*γ* and CD8 levels (Figure 5G,H) was achieved after treating with ScNPs‐PhotoCORM nanovaccine plus X‐ray irradiation, validating the in vivo adaptive anti‐tumor immunity activation induced by soft X‐ray triggered CO release.

Despite the enormous achievement in cancer immunotherapy, the immunotherapeutic efficacy is still limited by the immunosuppressive state of the deep‐seated solid tumors^[^ ^5^, ^6^ ^]^ due to the main infiltration of immunosuppressive M2 macrophages.^[^ ^7^ ^]^ Therefore, it is still a challenge to reverse the immunosuppressive TME for promoting cancer immune therapy. Recent studies suggested that high levels of ROS generation in tumor cells could further trigger the overexpression of inflammation in solid tumors.^[^ ^44^, ^45^, ^46^, ^47^ ^]^ And, as demonstrated in Figure 5A, the immunosuppressive TME can be reversed by promoting the pro‐inflammatory cytokine secretions of tumor necrosis factor *α* (TNF‐*α*) and IL‐6 (secreted by M1 macrophages) and suppressing the secretion of anti‐inflammatory cytokine of IL‐10 (marker of M2 macrophages).^[^ ^7^, ^48^ ^]^ Therefore, we expected that our developed nanovaccine with efficient ROS generation can also reverse the immunosuppressive TME.

To validate our assumption, the secretions of IL‐6, TNF‐*α* and IL‐10 were then evaluated. As presented in Figure 5D,F, the immunofluorescence images of the tumor slices revealed that IL‐6 and TNF‐*α* in control, X‐ray, ScNPs‐PhotoCORM, X‐ray plus ScNPs groups showed obvious no expression. In contrast, the expression levels of IL‐6 and TNF‐*α* were significantly increased (Figure 5I,K) in the tumors after treating with ScNPs‐PhotoCORM nanovaccine plus X‐ray. Meanwhile, the immunofluorescence intensity of anti‐inflammatory cytokine IL‐10 was sharply suppressed (Figure 5E,J) when treated with ScNPs‐PhotoCORM nanovaccine plus X‐ray. These findings demonstrate that the soft X‐ray mediated CO gas therapy can successfully activate the deep tissue anti‐tumor immunity response and further reverse the immunosuppressive TME.

### 2.5 In Vivo Systemic Anti‐Tumor Immune Responses Induced by Soft X‐Ray Sensitized CO Gas Therapy

**Figure 6:**
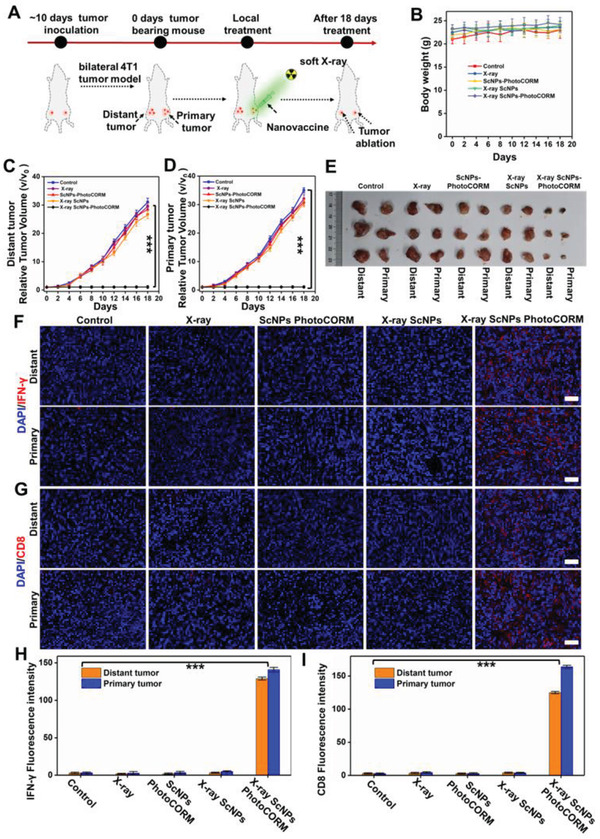
Systemic anti‐tumor immune responses activated by soft X‐ray triggered CO gas therapy in bilateral tumor model. A) Schematic illustration of the bilateral 4T1 tumor model for local CO therapy activated systemic anti‐tumor immune responses (only intratumor injection of ScNPs‐PhotoCORM nanovaccine, ScNPs, or PBS into the primary tumor and no treatment for distant tumor). B) The changes of body weight in different groups of bilateral 4T1 tumor‐bearing mice. C) Primary and D) distant tumor growth curves of the 4T1 tumor‐bearing mice after different treatments: Control; X‐ray irradiation; ScNPs‐PhotoCORM; X‐ray plus ScNPs; X‐ray plus ScNPs‐PhotoCORM in 18 days, ^***^ *P* < 0.001. E) Digital photographs of mice tumors removed from bilateral 4T1 tumor‐bearing mice after 18 days treatment. F,G) Immunofluorescence images of tumor slices acquired from different groups. Tumor slices were stained with F) IFN‐*γ*, G) CD8 anti‐body. H,I) Statistical data of the IFN‐*γ* and CD8 positive fluorescence intensity acquired from (F and G), respectively. ^***^ *P* < 0.001. All scale bars: 50 µm.

To shed more light in CO gas therapy induced systemic anti‐tumor immunoresponse, the bilateral 4T1 tumor‐bearing mice model was then constructed to further identify whether CO gas therapy could activate deep tissue systemic anti‐tumor immune responses after local treatment under X‐ray irradiation. The bilateral 4T1 tumors were cultured at the left and right flanks of mice and renamed as distant and primary tumors, respectively. As shown in **Figure** **6A**, the primary tumors were treated with PBS, X‐ray, ScNPs‐PhotoCORM, X‐ray plus ScNPs, X‐ray plus ScNPs‐PhotoCORM nanovaccine. Then the primary tumors in X‐ray, X‐ray plus ScNPs, X‐ray plus ScNPs‐PhotoCORM groups were irradiated with soft X‐ray light for 10 min every day. No obvious weight losses were observed in all groups after 18 days of treatment (Figure 6B). As expected, the growth rate of the primary tumor with ScNPs‐PhotoCORM plus X‐ray treatment showed noticeable inhibition compared with the PBS, only X‐ray irradiation, ScNPs‐PhotoCORM, X‐ray plus ScNPs groups, indicating the effective local CO gas therapy (Figure 6D,E). More interestingly, the distant tumor in the ScNPs‐PhotoCORM plus X‐ray group (Figure 6C,E) was also obviously inhibited, which was mainly attributed to the local therapy activated systemic anti‐tumor immune response. HE (Figure S16, Supporting Information) analysis of the primary and distant tumor also showed obvious apoptotic and necrotic tumor cells in the ScNPs‐PhotoCORM plus X‐ray treatment group. To further reveal the systemic anti‐tumor immune responses, both the primary and distant tumors were then stained with IFN‐*γ* and CD8 anti‐bodies. As shown in Figure 6F–I, compared with the other control groups, the ScNPs‐PhotoCORM plus X‐ray treatment showed remarkably increased IFN‐*γ* and CD8 expression levels in both primary and distant tumors, indicating the successful activation of systemic anti‐tumor immune responses after CO gas mediated local tumor therapy. To further evaluate the in vivo biotoxicity of the ScNPs‐PhotoCORM nanovaccine, we have performed the blood biochemistry tests. As shown in Figure S17, Supporting Information, blood biochemistry data indicated the high biocompatibility of the nanovaccine. All these results revealed that the soft X‐ray induced CO gas therapy can effectively activate deep tissue adaptive anti‐tumor immunity and reverse the immunosuppressive TME, subsequently resulting in systemic anti‐tumor immune responses.

## 3 Conclusion

In conclusion, a novel low dose soft X‐ray stimulated lanthanide scintillator‐based CO releasing nanovaccine was designed for synergetic gas and anti‐tumor immunotherapy for the first time. Benefiting from the greatly improved (≈7.5 times) XEF properties, unprecedented deep tissue (>5 cm) CO release was achieved under soft X‐ray light irradiation for in vivo deep tissue tumor therapy, breaking through the barrier of the depth limitation encountered by the traditional NIR light. Significantly, the cascade reaction triggered by CO gas therapy further provoked the generation of ROS, resulting in effective ICD. Meanwhile, the enhanced oxidative stress further opened up the inflammation signaling pathway and reversed the immunosuppressive TME, subsequently activating the anti‐tumor immune responses. More interestingly, both the tumor growths of the primary and distant tumors were significantly suppressed by local gas therapy activated systemic anti‐tumor immunoresponse. Therefore, our study opens up a new possibility of designing nanovaccine by integrating soft X‐ray activated lanthanide scintillator with PhotoCORM to realize deep tissue gas therapy, activate systemic anti‐tumor immunoresponse, and reverse immunosuppressive TME with breaking the barrier of the penetration depth.

## Conflict of Interest

The authors declare no conflict of interest.

## Supporting information

Supporting Information

## Data Availability

Research data are not shared.
