# Supplementary material for: Low Dose Soft X‐Ray Remotely Triggered Lanthanide Nanovaccine for Deep Tissue CO Gas Release and Activation of Systemic Anti‐Tumor Immunoresponse
Source: Adv Sci (Weinh). 2021 Apr 8;8(12):2004391. doi: 10.1002/advs.202004391 (PMC8224418; doi:10.1002/advs.202004391)
Supplement: Supplementary file 1 — Supporting Information [file ADVS-8-2004391-s001.pdf]

© 2021 Wiley-VCH GmbH

ADVANCED SCIENCE
Open Access

Supporting Information

for *Adv. Sci.,* DOI: 10.1002/advs.202004391

Low Dose Soft X-ray Remotely Triggered Lanthanide Nanovaccine for Deep Tissue CO Gas Release and Activation of Systemic Anti-Tumor Immunoresponse

Youbin Li, Mingyang Jiang, Zhiming Deng,Songjun Zeng, and Jianhua Hao,**

Supporting Information

Low Dose Soft X-ray Remotely Triggered Lanthanide Nanovaccine for Deep Tissue CO Gas Release and Activation of Systemic Anti-Tumor Immunoresponse

Youbin Li,[1] Mingyang Jiang,[1] Zhiming Deng,[1] Songjun Zeng[1,]* and Jianhua Hao[2,]*

Prof. S. J. Zeng, Y. B. Li, M. Y. Jiang, Z. M. Deng,

[1]Synergetic Innovation Center for Quantum Effects and Application, Key Laboratory of Low-dimensional Quantum Structures and Quantum Control of Ministry ofEducation, Key Laboratory for Matter Microstructure and Function of Hunan Province, School of Physics and Electronics, Hunan Normal University, Changsha, 410081,PR China

E-mail: **songjunz@hunnu.edu.cn**

Prof. J. H. Hao,

[2]Department of Applied Physics, The Hong Kong Polytechnic University, Hong Kong, P.R. China

E-mail:**jh.hao@polyu.edu.hk**

METHODS

Chemicals and Materials. Rare earth YCl_3•$6H_2O$ (99.99%), $LuCl_3$•$6H_2O$ (99.99%), $GdCl_3$•$6H_2O$ (99.99%), and$TbCl_3$•$6H_2O$ (99.99%) were purchased from QingDa elaborate Chemical Reagent Co. Ltd (Shandong). Oleic acid (OA, 90%), 1-Octadecene (ODE, 90%), NH_4F,NaOH, CH_3OH (99.5%), anhydrous alcohol, manganese pentacarbonyl bromide,2,2' bipyridine, diethyl ether,THF, Ag(CF_3SO_3),DSPE-PEG(2000) carboxylic acid (1,2-distearoyl-snglycero-3-phosphoethanolamine-N-[carboxy(polyethyleneglycol)-2000), and all other reagentswereobtained from Sinopharm Chemical Reagent Co., Ltd. China. All chemical reagents were analytical grade and used without further purification.

Synthesis of $NaLuF_4$:20Gd,xTb (x=15, 20, 30)Core Nanoparticles. The $NaLuF_4$:20Gd, xTb (x=15, 20, 30) core nanoparticles were preparedby a modified high-temperature co-precipitation method according to our previous report.[s1]$LuCl_3$•$6H_2O$, $GdCl_3$•$6H_2O$, and$TbCl_3$•$6H_2O$with designed mole ratio were added into a mixture of OA (12 mL) and ODE (30 mL) solution. The solution was heated to 160℃for 1h, and then cooled down to 90 ℃.Then,a mixture solution of NaOH(0.1 g) in methanol (10 mL) and NH_4F (0.148 g) in methanol (20 mL)was added and kept for 1 h at room temperature.The solution was then heated to 305 ℃ and maintained for 1 h.After cooling down to room temperature, the solution was centrifuged and washed with ethanol for three times. Finally, the as-prepared nanoparticles were re-dispersed in cyclohexane for further use. All the reaction procedureswere under argon gas protection.

Synthesis of $NaLuF_4$:20Gd, xTb (x=15, 20, 30)@$NaLnF_4$(Ln=Y, Lu, Gd) Core-ShellNanoparticles. In a typicalprocedure, 1 mmolof $LnCl_3$•$6H_2O$ and the as-prepared core nanoparticles (1 mmol)were added into the three-neck flask containing OA (12 mL) and ODE

(30 mL). Themixture washeated to 160 ℃ for 1h, and then cooled down to 90 ℃. After that, a mixture solution of NaOH (0.1 g) in methanol (10 mL) and NH_4F (0.148 g) in methanol (20 mL)was added and kept for 1 h at room temperature. Then, the mixture was heated to 305 ℃ for 30 min and cooled down to roomtemperature.The resultant nanoparticles were centrifuged and washed with ethanol three times.The final productswere re-dispersed in 5 mL of cyclohexane for further use.

Surface Modification of the $NaLuF_4$:20Gd,20Tb@$NaLuF_4$Core-Shell ScintillatorsNanoparticles (ScNPs) with DSPE-PEG(2000)-COOH. In a typical experiment,[s2] 20 mg of ScNPsin 5 mL of chloroformwere added into 5 mL ofchloroform solution containing 50 mg of DSPE-PEG(2000)-COOH. After gently stirring for 30 min, a rotary evaporator was used to evaporate the excess chloroform in water bath at 30 °C.Thefinal products were obtained by centrifugation and re-dispersed in 2 mL of water for further use.

Preparation of dicarbonyl(bipyridine)bis(triphenylphosphine)manganese(I) trifluoromethylsulfonate: Mn(bpy)$(CO)_2(PPh_3)_2$](CF_3SO_3). In a typical process,[s2]a mixture of diethyl ether solution (50 mL) containing manganese pentacarbonyl bromide (0.275 g) and 2,2' bipyridine (0.172 g)was refluxed for 4 h at room temperature. After cooling the reaction solution to -40 °C, an orange solid Mn(bpy)$(CO)_3$Br was precipitated and collected. Then, Mn(bpy)$(CO)_3$Br (0.188 g) and Ag(CF_3SO_3) (0.141 g) were added into the flask containing 50 mL of dry THF for further reflux for another 6 h. Then, the reactedsolution was filtered through a fine porosity sintered glass frit to remove the insoluble AgCl. After that, triphenylphosphine (1.31g) was added into the filtered solution and refluxed for 18 h.Finally, the remaining THF was evaporated in vacuum to form dark orange [Mn(bpy)$(CO)_2(PPh_3)_2$](CF_3SO_3).

Preparation of ScNPs-PhotoCORMNanovaccine. For loading photo-responsive carbon monoxide releasing moiety (PhotoCORM) to form ScNPs-PhotoCORM nanovaccine,the prepared $[Mn(bpy)(CO)_2(PPh_3)_2](CF_3SO_3)$ (50 mg) was added into 2 mL of the ScNPs-PEG solution (1% acetonitrile in water) and kept stirring for 24 h in the dark. The resulted nanovaccines were then collected by centrifugation and washed with acetonitrile.

Measurement of CO Release Concentration. The soft X-ray triggered CO release levels were evaluated by a myoglobin assay method according to the previous report.[s3]First, the hemoglobin (MP Biomedicals, 4.2μM concentration)wascompletelydissolved in phosphate buffered saline (10 mM pH=7.4 PBS).Then, excess sodium dithionite was added into the solution under a nitrogen atmosphere protection. After that, the ScNPs-PhotoCORM nanovaccine (10μg/mL)was added into the solution for further detection under soft X-ray irradiation. The CO release concentration and hemoglobin (Hb)-to-carboxyhemoglobin(HbCO) conversion percentage (x)was measured according to the Beer-Lambert law by the following formula[s3] :

$$\frac{I_{410\ nm}}{I_{430\ nm}} = \frac{\varepsilon_{Hb410nm}(1-x) + \varepsilon_{HbCO410\ nm}x}{\varepsilon_{Hb430nm}(1-x) + \varepsilon_{HbCO430\ nm}x}$$

Where$I_{410\ nm}$ and $I_{430\ nm}$ represent the intensities of the collected absorption spectra, ε_{Hb410}, $\varepsilon_{HbCO410}$, ε_{Hb430}, and$\varepsilon_{HbCO430}$represent the molar extinction coefficients of Hb and HbCO at 410 and 430 nm, respectively.

According to the previous literatures,[s2,s3]the ε_{Hb410}-$\varepsilon_{HbCO410}$, ε_{Hb430}-$\varepsilon_{HbCO430}$, ε_{Hb410}, and ε_{Hb430} were measured to be -442.4 $mM^{-1}cm^{-1}$, 216.5 $mM^{-1}cm^{-1}$, 304 $mM^{-1}cm^{-1}$, and 528.6 $mM^{-1}cm^{-1}$, respectively. Then the concentration of CO (C_{co}) can be evaluated as the following formula.

$$C_{co} = C_{Hb} \times x = \frac{I_{410nm}528.6 - I_{430\,nm}304}{I_{410nm}216.5 + I_{430\,nm}424.4} C_{Hb}$$

Where C_{co} and C_{Hb} represent the released CO concentration and Hb concentration, respectively.

Calculation of the Fluorescence Resonance Energy Transfer (FRET)Efficiency. The FRET efficiency was evaluatedaccording to the following formula[s4]:

$$E = 1 - \frac{I}{I_0}$$

where I and I_0 are the fluorescence intensities of the donors inthe presence of acceptors (PhotoCORM), and in the absence of acceptors, respectively.

Characterizations. ThepowderX-ray diffraction (XRD) data of the as-preparedsampleswere detected with a Rigaku D/max 2500 system operating at 40 kV and 250 mA. Transmission electron microscopy (TEM) images were performed on a TEM(FEI Tecnai F20) operated at an acceleration voltage of 200 kV. The X-ray excited radio-fluorescence(XEF) spectra were recorded by aZolixOmniFluo-Xrayfluorescence spectrometer equipped by an X-ray excitation source(MAGPRO) with tunable tube voltage from 4-70 kV.The UV-Vis absorbance spectra of the nanocrystals were conducted on a spectrophotometer system (UV-1800, Hunan Sino-Jewell Electronics Co.Ltd.).

Tumor Animal Models:8×10^6 breast cancer (4T1) cells were subcutaneously injected into the BALB/c mice to obtain the 4T1 tumor-bearing mice models. The 4T1 tumor-bearing mice were used for X-ray bioimaging and soft X-ray induced CO-sensitized gastherapy. The animals used in the experiments have complied with the institutional animal use of Hunan Normal University approved by the Laboratory Animal Center of Hunan Province.

***In Vivo*Soft X-ray Activated Optical Bioimaging**. The ScNPs-PhotoCORMnanovaccines (3 mg/mL) were first subcutaneously injected into the tumor site of the tumor-bearing mice for X-ray activated bioimaging by a multi-modal *in vivo* imaging system (Bruker In Vivo FX

Pro)equipped with a detecting CCD (ML4002, Finger Lakes Instrumentation, USA).Then, X-ray activated optical imaging was performed with different irradiation times (1, 2, 3, 5 min) and various excitation tube voltages (30, 35, 40, 45 kVp).

***In Vitro*Soft X-ray Induced OpticalBioimaging**.*In vitro*soft X-ray induced bioimagingwas performed by using the same system. Different concentrations of ScNPs-PhotoCORMnanovaccines in 96 well tubes were used for soft X-ray induced bioimaging under different irradiation times (1, 2, 3, 5 min) andvarious excitation tube voltages (30, 35, 40, 45 kVp).ScNPs-PhotoCORM (3 mg/mL) nanovaccinein the 96 well tubes was further used for depth-dependent optical bioimaging under soft X-ray irradiation.

***In Vitro*Cytotoxicity and Gas Therapy of Cancer Cells**.*In vitro* cell viability of the ScNPs-PhotoCORMnanovaccinewas evaluated by using a 3-(4,5-dimethylthiazol-2-yl)-2-5-diphenyl-tetrazolium bromide (MTT) proliferation assay method. The 4T1 cells were first cultured in a 96-well microplate and kept at 37 ℃ under 5% CO_2 for 3 h. Then the cell culture medium was substituted with Dulbecco’s Modified Eagle Medium (DMEM) solution including 10% fetal bovine serum and 1% penicillin and streptomycin. For cell toxicity tests, the cells were then treated with different concentrations of ScNPs-PhotoCORMnanovaccine for 24 h at 37 °C. Finally, the cell viabilities were tested by using MTT method. For *in vitro*therapy of cancer cells, the cells were divided into five groups inrandom for various treatments: #1. Control group with PBS; #2. Only X-ray irradiation; #3.ScNPs-PhotoCORM (0.5 mg/mL, 100 μL) without X-ray irradiation; #4.ScNPs (0.5 mg/mL, 100 μL) with X-ray irradiation; #5.ScNPs-PhotoCORM (0.5 mg/mL, 100 μL)group with softX-ray irradiation (Dose rate:490 μGy/s).The cells were then cultured at 37 °C for 24 h. Finally, the cell viabilities were tested referring to the MTT method.

The *in vitro*depth dependentCOgas therapy of the upconversion nanoparticles (UCNPs, $NaYbF_4$:Tm@$NaGdF_4$ core-shell nanoparticles)based PhotoCORM(UCNPs-PhotoCORM) andScNPs-PhotoCORMnanovaccineswas demonstrated by covering with different thicknesses of pork tissueon the samples under various light excitation (UCNPs-PhotoCORM: 980 nm laser, 100 mW/cm^2, 10 min; ScNPs-PhotoCORM: soft X-ray irradiation). The cell viability was measured by using the same method.

***In Vitro* Oxidative Stress Detection.**4T1 cells were first treated with PBS, only X-ray irradiation, ScNPs-PhotoCORM (30 μg/mL, 10 μL), ScNPs (30 μg/mL, 10 μL), and ScNPs-PhotoCORM (30 μg/mL, 10 μL) and incubated for 12 h. And, the ScNPs and ScNPs-PhotoCORM groups were irradiated under soft X-ray light for 5 min and incubated overnight.Then the 4T1 cells were stained with 2',7'-dichlorodihydrofluorescein (DCFH-DA) and 4',6-diamidino-2-phenylindole (DAPI) for 0.5 h. The fluorescence images were then acquired by using the confocal fluorescence microscope.

***In Vitro*Damage-associated Molecular Patterns (DAMPs) Detection.**4T1 cells were first treated with PBS,only X-ray irradiation, ScNPs-PhotoCORM(30μg/ml, 10 μL), ScNPs (30 μg/ml, 10 μL), and ScNPs-PhotoCORM (30μg/ml, 10 μL) and incubated for 12 h. Then, theScNPs and ScNPs-PhotoCORMtreated groups were irradiated under soft X-ray light for 5 min. Finally, the culture medium was collected for further detection. Extracellular secretion of adenosine triphosphate(ATP) was detected by using the commercially available ATP assay kit according to the manufacturer's protocols.The extracellular released high mobility group box 1(HMGB-1)was detected by using the HMGB-1 enzyme-linked immunosorbent assay (ELISA) Kit according to the manufacturer's protocols.For intracellular calreticulin(CRT) detection, the cells with different treatments were stained with anti-calreticulin for 12 h, and then theimmunofluorescence images were taken on a confocal fluorescence microscope.

***In Vivo* Gas-Sensitized Anti-Tumor Therapy and CO Content Measurement**.For *in vivo* X-ray induced gas therapy, the 4T1 tumor-bearing mice were divided into five groups (3 mice per group)at random including#1. Control treated with PBS; #2. X-ray irradiation without injection; #3.ScNPs-PhotoCORM without X-ray irradiation;#4.ScNPs with X-ray irradiation; #5.ScNPs-PhotoCORM group with X-ray irradiation (ScNPs:3 mg/mL, 125 μL; ScNPs-PhotoCORM: 3 mg/mL, 125 μL).And, the ScNPs-PhotoCORM (3 mg/mL, 125 μL) was intravenously injected into the mice every fifth day for nearly3times in 20 days. The tumor size and weight of the mice were recorded.After various treatments, the tumor tissues were quickly collected from the tumor-bearing mice for further CO detection by using the endogenous COassay kit (A101-2).The CO assay kit was purchased from Nanjing Jiancheng Bioengineering Institute.

The *in vivo* depth dependentgas therapy based on the UCNPs-PhotoCORM(3 mg/mL, 125 μL; 980 nm laser power density: 0.5 W/cm^2; 980 nm laser irradiation time: 10 min every day) and SCNPs PhotoCORMnanovaccines(3 mg/mL, 125 μL; X-ray irradiation time: 10 min every day)was demonstrated by covering with different thicknesses of pork tissue on the tumor.

For *in vivo* X-ray induced gas therapy of bilateral 4T1 tumor-bearing mice, the bilateral 4T1 tumor-bearing mice were constructed at the left and right flanks of mice and renamed as distant and primary tumor, respectively and divided into five groups (3 mice per group) including #1. Control group with PBS injection; #2. Only X-ray irradiation; #3. ScNPs-PhotoCORM without X-ray irradiation; #4. ScNPs plus X-ray irradiation; #5. ScNPs-PhotoCORM group plus X-ray irradiation (ScNPs:3 mg/mL, 125 μL; ScNPs-PhotoCORM: 3 mg/mL, 125 μL). Then, the ScNPs and ScNPs-photoCORMnanovaccines (3 mg/mL, 125 μL) wereintratumorally injected into the primary tumor in the # 3, 4, 5 groups. The tumor size and weight of the mice were recorded.

***In Vivo*HbCOand HIF-1αContent Detection**. For *in vivo*HbCOconcentration measurement, the 4T1 tumor-bearing mice treated with PBS, and X-ray plus ScNPs-PhotoCORMnanovaccines for 8 days and 16 days were collected for further HbCO detection by using theHbCOassay kit (GOY-E0002379120). For HIF-1αmeasurement, the tumor slices were stained with fluorescence labeled HIF-1α antibody for further detection.

Immunofluorescence Tests. The tumors after different treatments were dissected from the mice after 20 days treatment.Then the tumor slices were stained with fluorescence labeled CD8, TNF-α, IL-6, HMGB1, CRT, IL-10, andIFN-γantibodiesfor detection.The immunofluorescence images were taken on a confocal fluorescence microscope.

For immunofluorescence testsof bilateral 4T1 tumor-bearing mice, the primary and distant tumorsafter different treatments were dissected after 14 days treatment. Then the tumor slices were stained with fluorescence labeled CD8 and IFN-γ antibodiesfor detection. The immunofluorescence images were taken on a confocal fluorescence microscope.

Tumor Biopsies. For hematoxylin and eosin(HE)andTerminal deoxynucleotidyltransferasedUTP nick end labeling(TUNEL)staining, the tumors of 4T1 tumor-bearing mice and bilateral 4T1 tumor-bearing miceafter different treatments were dissected.And then the isolated tumors were stained with HE and TUNEL for histological test.

***In Vivo*Safety Evaluation.**For *in vivo* safety study, theBALB/cmice wereinjected with PBS, ScNPs-PhotoCORM (3 mg/mL, 125 μL) for 1 day and 3 days.The blood samples were collected for further biochemistry tests.And the hepatic function markers ofalanine transferase (ALT), aspartate transaminase (AST), alkaline phosphatase (ALP) and renal function marker of blood urea nitrogen (BUN) were evaluated.

Statistical Analysis. All the results werepresented as the mean ± standarddeviation (S.D.). All statistical comparisons between groups were analyzed by one-way ANOVA method using

SPSS software. The criterion for statistical significance was taken as ***$p < 0.001$, **$p < 0.01$, *$p < 0.5$.

Supplement References:

[s1] Y.B.Li,X.L.Li,Z.L.Xue,M.Y. Jiang,S.J.Zeng,J.H.Hao,*Adv. Healthcare Mater.* **2017**, *6*, 1601231.

[s2]A.E.Pierri, P.J.Huang, J.V.Garcia, J.G.Stanfill, M.Chui, G.Wu, N. F. Zheng, P.C.Ford, *Chem. Commun*. **2015**, *51*, 2072.

[s3]Q.J.He, D.O. Kiesewetter, Y.Qu, X.Fu, J.Fan, P.Huang, Y.J.Liu, G.Z.Zhu, Y. Liu, Z.Y.Qian, X.Y. Chen, *Adv. Mater*. **2015**, *27*, 6741.

[s4] A. F. G. Monte, G.Azevedo, A. F. Reis, *Luminescence***2020**, *35*, 667.

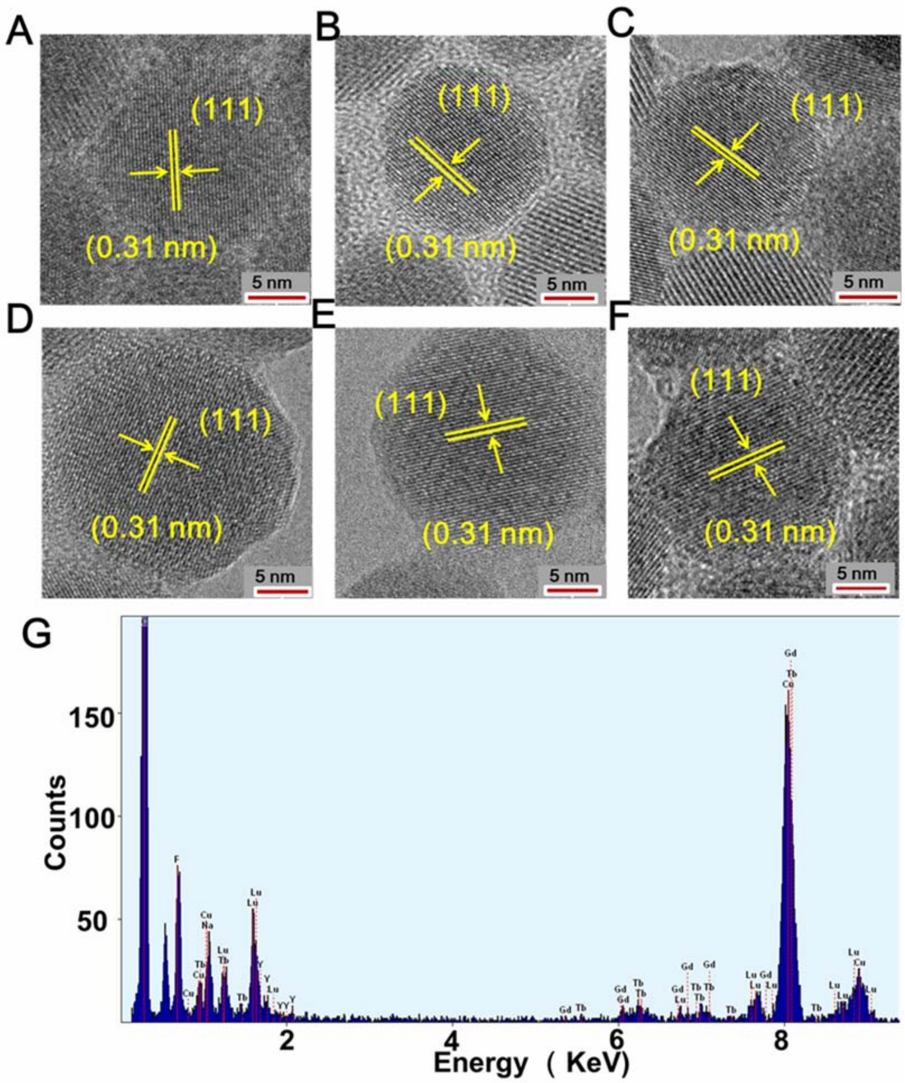

Figure S1. (A)-(C)High resolution (HR) TEM images of the as-prepared core (Figure 1B-1D) nanoparticles, respectively. (D)-(F)HRTEM images of the corresponding core-shell(Figure 1F-1H) nanoparticles, respectively. (G) EDS pattern of the as-prepared $NaLuF_4$:20Gd/20Tb@$NaYF_4$core-shell nanoparticles.

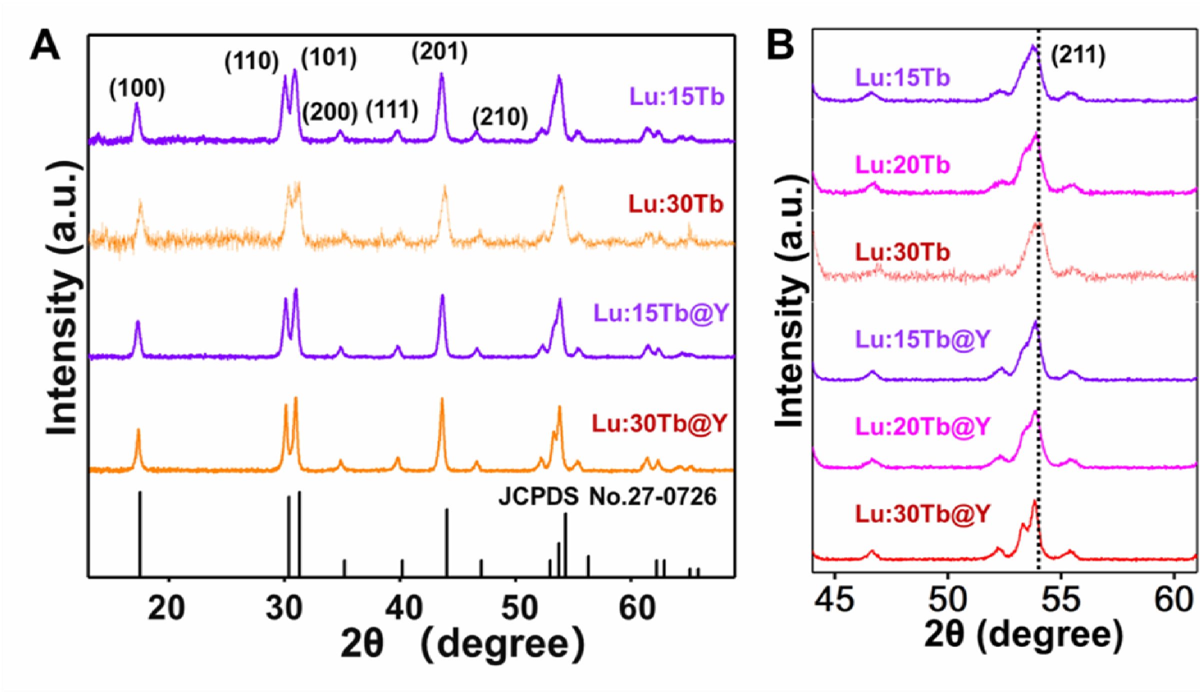

Figure S2. (A)XRD patterns of the as-prepared $NaLuF_4$:Gd,xTb (x=15, 30) core and $NaLuF_4$:Gd,xTb@$NaYF_4$(x=15,30) core-shell nanoparticles. (B) The amplified XRD patterns of the as-prepared $NaLuF_4$:Gd,xTb (x=15, 20, 30) core and $NaLuF_4$:Gd,xTb@$NaYF_4$ (x=15, 20, 30) core-shell nanoparticles.

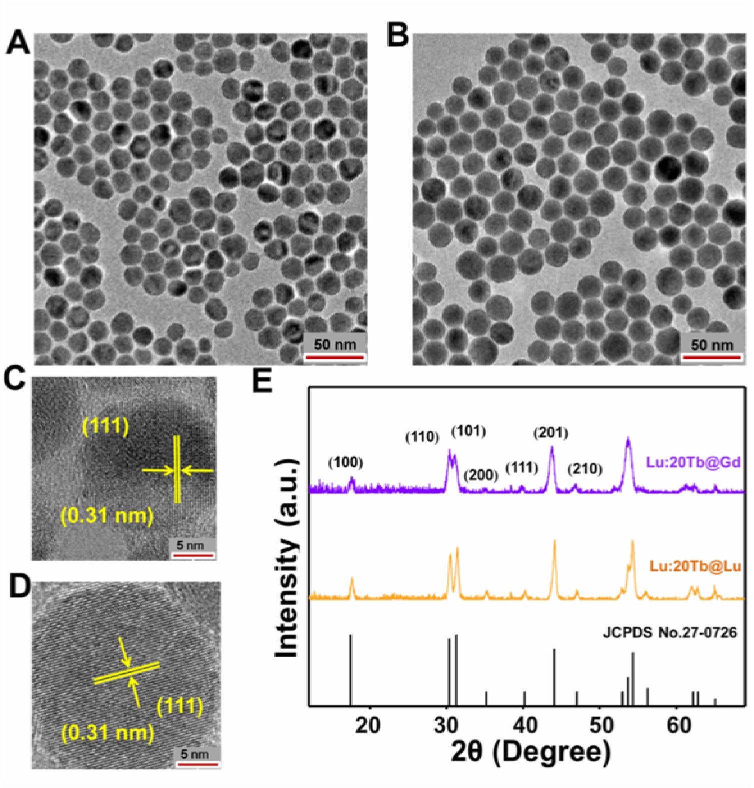

Figure S3. (A) and (B) TEM images of $NaLuF_4$:20Gd,20Tb@$NaGdF_4$ and $NaLuF_4$:20Gd,20Tb@$NaLuF_4$ core-shell nanoparticles, respectively. (C) and (D) HRTEM images of the as-prepared core-shell nanoparticles taken from(A) and (B), respectively.(E) XRD patterns of the as-synthesized $NaLuF_4$:Gd,20Tb@$NaLnF_4$(Ln=Gd, Lu) core-shell nanoparticles.

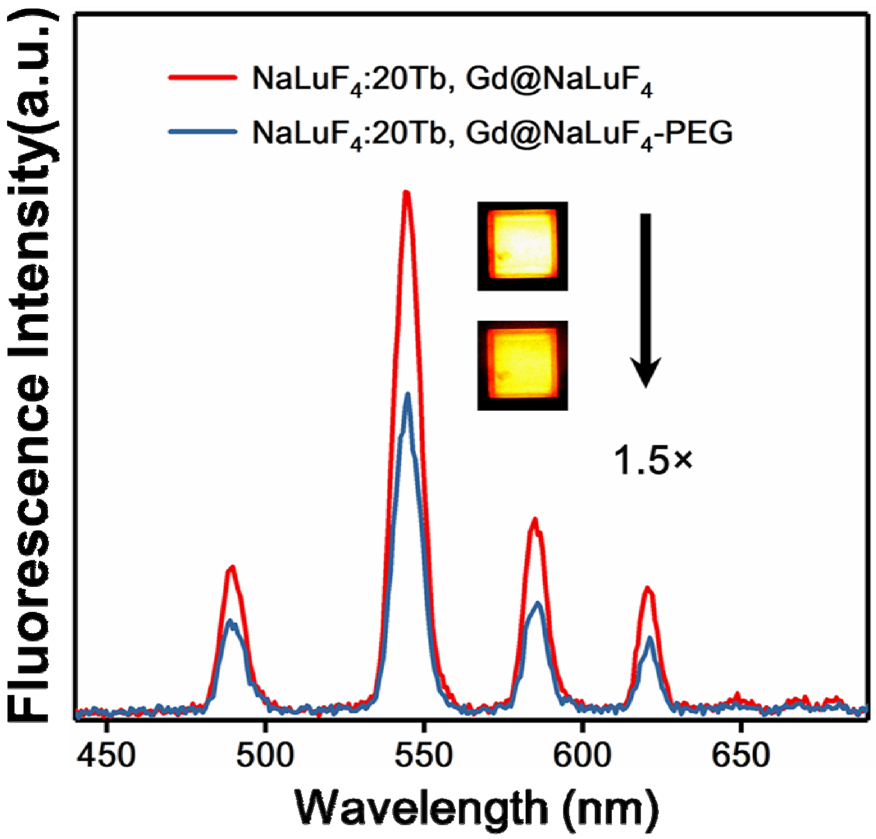

Figure S4. XEF spectra of the as-synthesized $NaLuF_4$:Gd,20Tb@$NaLuF_4$ and PEG-modified $NaLuF_4$:Gd,20Tb@$NaLuF_4$ core-shell nanoparticles,the inset picturesdenote the corresponding *in vitro*phantom fluorescence images under soft X-ray irradiation.

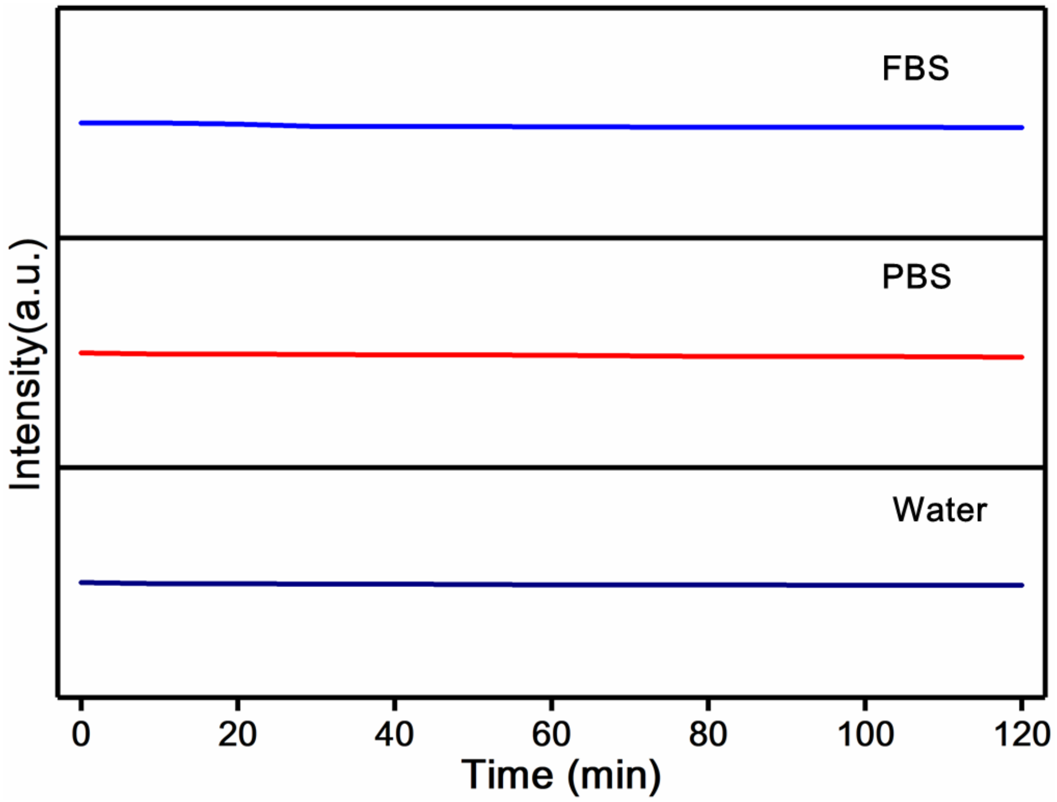

Figure S5. Photostability curves of PEG-modified $NaLuF_4$:Gd,20Tb@$NaLuF_4$ core-shell nanoparticles in various biological media under X-ray irradiation for 2 h.

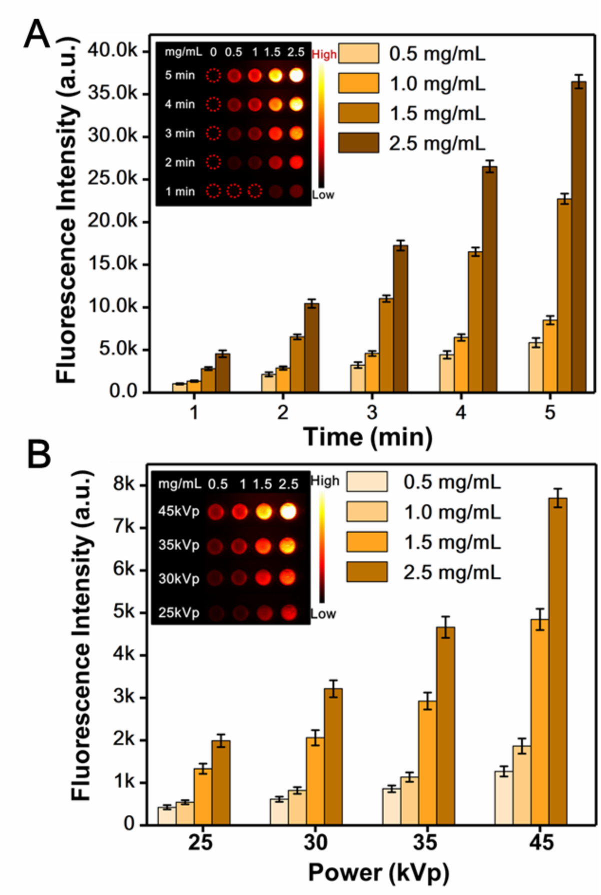

Figure S6. (A) Time-dependent fluorescence intensity of different concentrations of ScNPs-PhotoCORMnanovaccine, the inset shows the corresponding *in vitro*phantom fluorescence images under X-ray irradiation (Tube voltage:45kVp). (B) Power-dependent fluorescence images of different concentrations of ScNPs-PhotoCORMnanovaccine with X-ray irradiation, the inset shows the corresponding *in vitro*phantom fluorescence images under X-ray irradiation with various voltages.

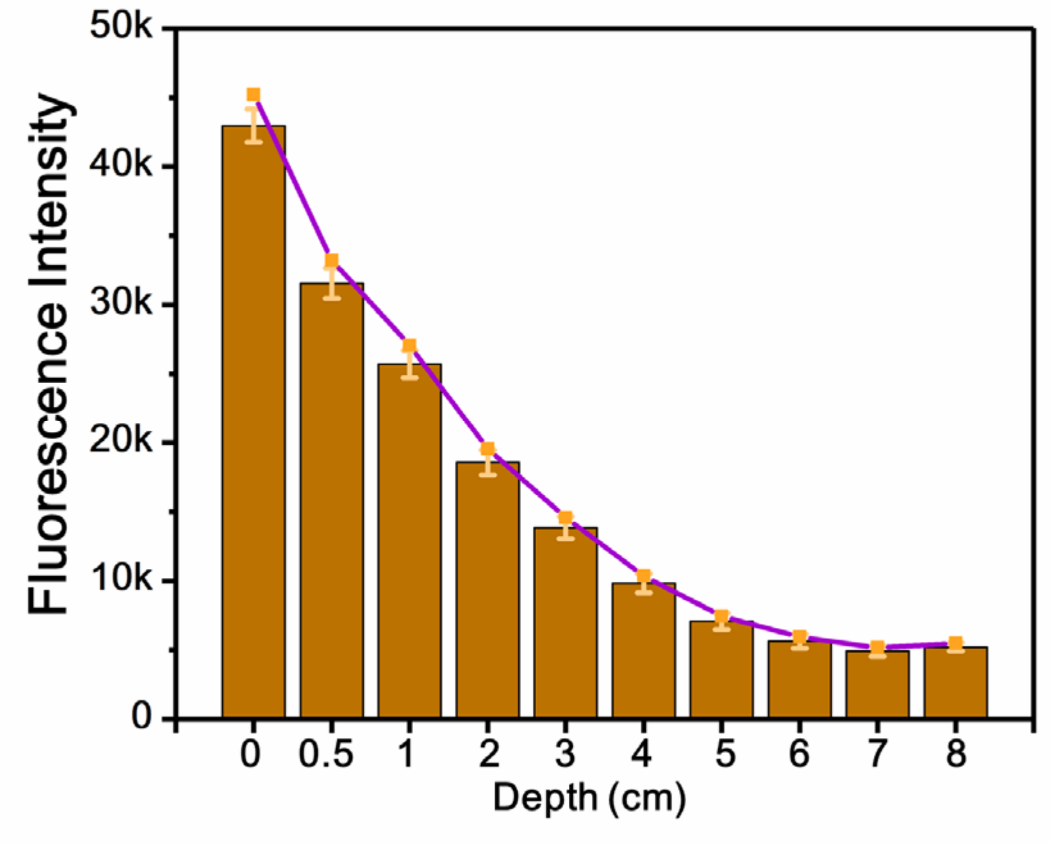

Figure S7. The corresponding fluorescence intensity of Figure 2Funder soft X-ray irradiation (Tube voltage:45kVp)

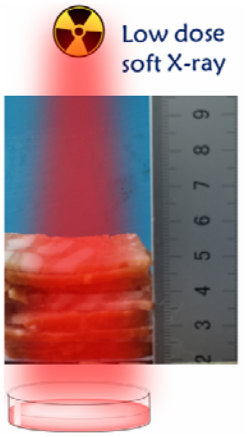

Figure S8. Schematic illustration of the depth-dependent CO release detection *via*covering different thicknesses of pork tissue on the solutionupon soft X-ray irradiation.

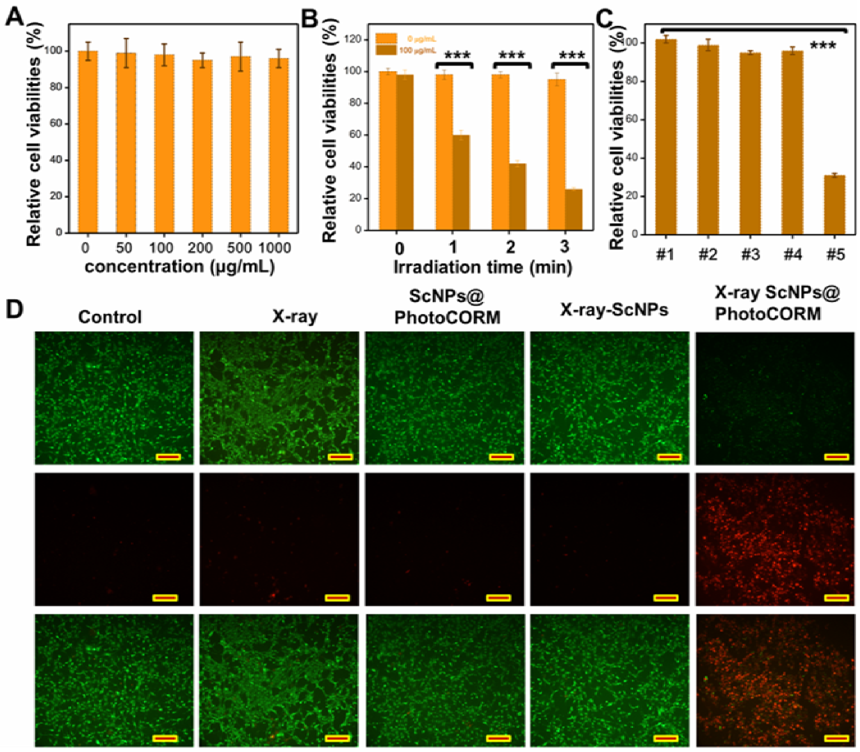

Figure S9. (A) The cell viability of 4T1 cells treated with different concentrations of ScNP-PhotoCORMnanovaccine. (B) The cell viabilities of 4T1 cells treated with 0μg/mL and 100 μg/mL of ScNP-PhotoCORMnanovaccine under different time of X-ray irradiation. $^{***}P<0.001$. (C) *In vitro* cell viabilities of 4T1 cells dealt with different treatments. (#1.Control; #2.X-ray irradiation; #3.ScNPs-PhotoCORM; #4.X-ray and ScNPs; #5.X-ray and ScNPs-PhotoCORM), $^{***}P<0.001$. (D) The confocal fluorescence images of 4T1 cells treated with live (stained by Calcein-AM, green fluorescence) and death (stained with PI, red fluorescence) staining. Scale bar: 400 μm.

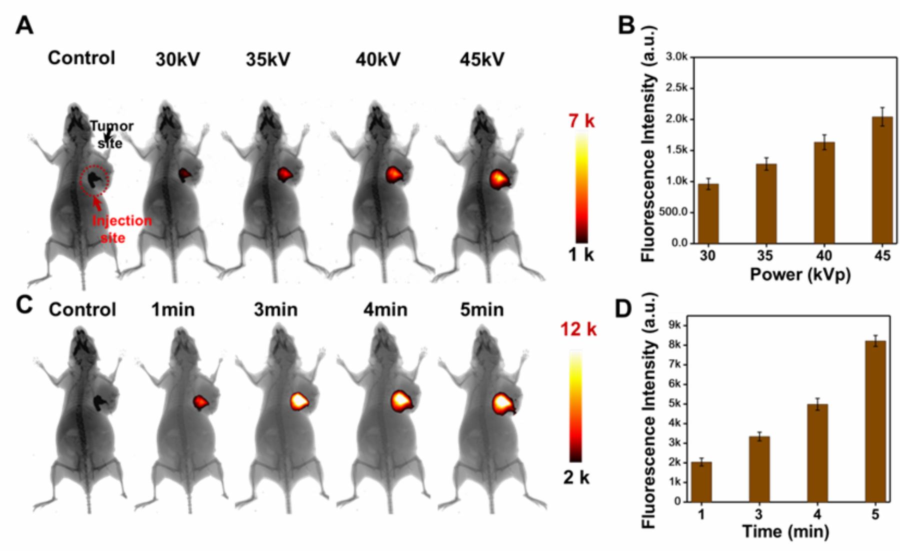

Figure S10. (A) The *in vivo* fluorescence images of tumor-bearing mice with subcutaneous injection ofScNPs-PhotoCORMnanovaccine in the tumor site under X-ray irradiation for 1 minwith different voltages and (B) the corresponding fluorescence intensity. (C) The *in vivo* fluorescence images of tumor-bearing mice with subcutaneous injection ofScNPs-PhotoCORMnanovaccine in the tumor site under different X-ray irradiation (45 kVp)times and (D) the corresponding fluorescence intensity.

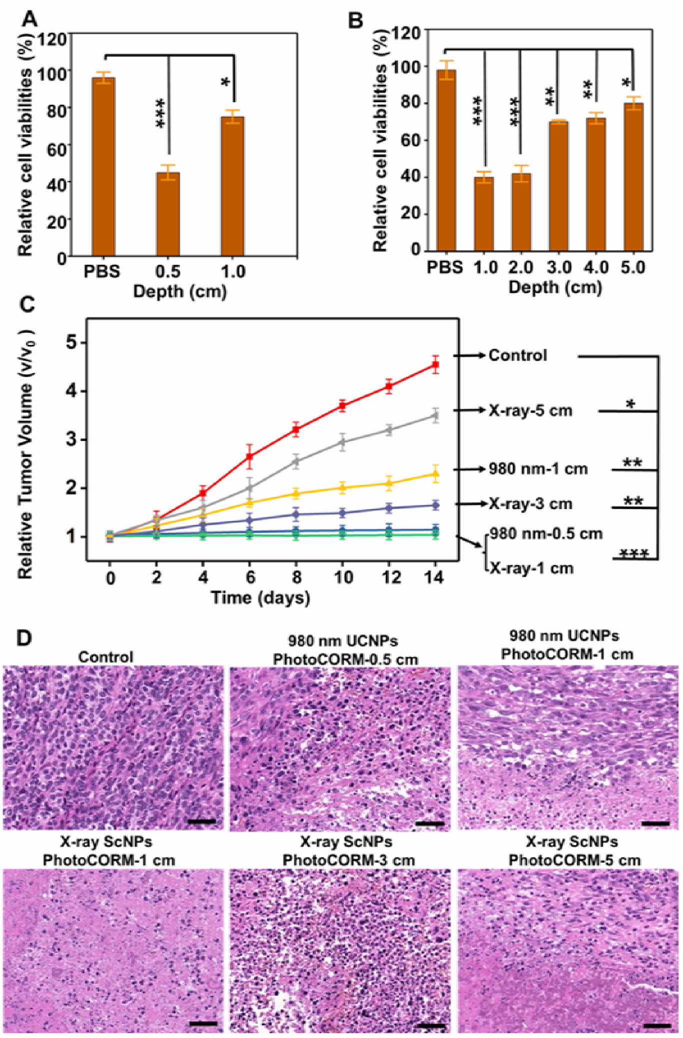

Figure S11.(A) Cell viabilities of 4T1 cellstreated with PBS and 100 μg/mL of UCNPs-PhotoCORMnanovaccineand covered with different thicknesses of pork tissue under 980 nm laser irradiation. (B) Cell viabilities of 4T1 cells treated with PBS and ScNPs-PhotoCORMnanovaccineand covered with different thicknesses of pork tissue under soft X-ray irradiation. (C) The tumor growth curves of the 4T1 tumor-bearing mice after various treatments. (D) HE stained images of tumor slices collected from different test groups. Scale bar: 40μm.***p < 0.001, **p < 0.01, *p < 0.5.

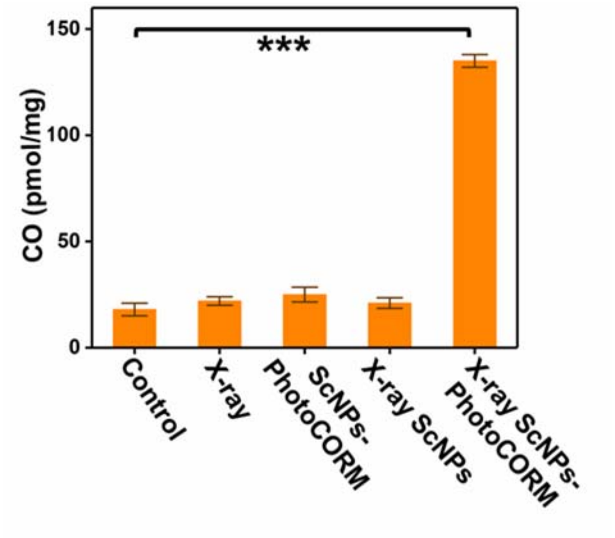

Figure S12.CO contents intumor tissues after various treatments. ***p < 0.001.

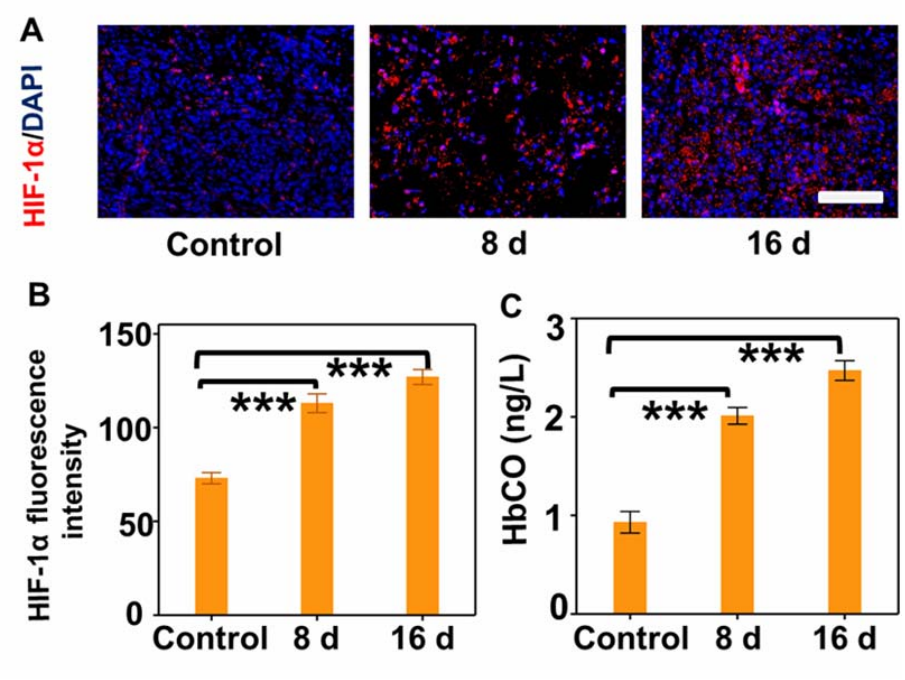

Figure S13.Immunofluorescence images of tumor slices obtained from tumor-bearing mice treated with PBS, X-ray plus ScNPs-PhotoCORMnanovaccine for 8 days and 16 days, respectively. The tumor slices were stained with HIF-1α antibody. (B)Statistical data of the HIF-1αpositive fluorescence intensity acquired from (A).(C)HbCOcontents in tumor tissues after various treatments. ***$p < 0.001$. Scale bar: 40 µm.

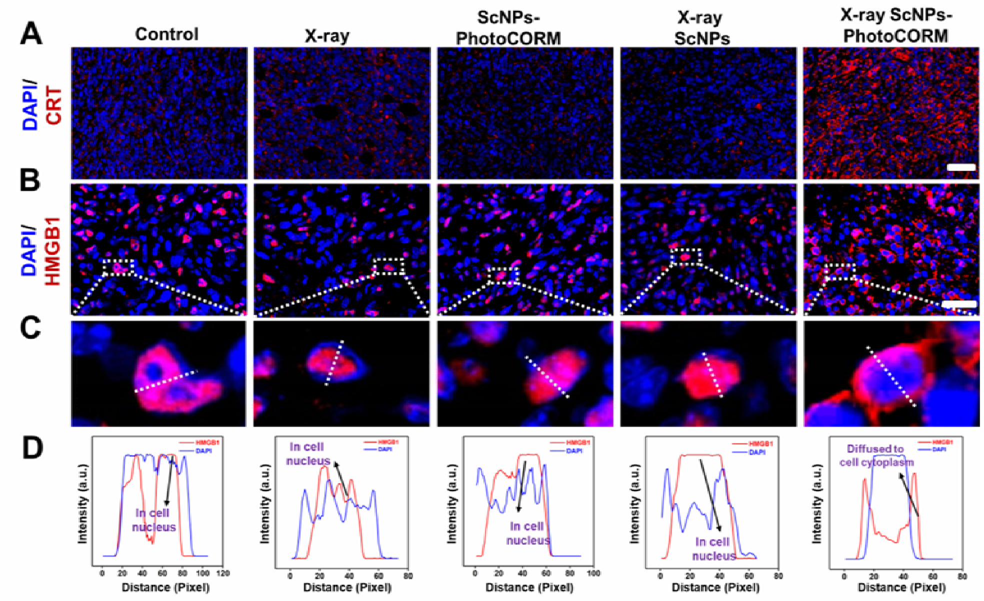

Figure S14.(A)and (B)Immunofluorescence images of tumor slices obtained from tumor-bearing mice with various treatments. Tumor slices were stained with (A) CRT, (B) HMGB1 antibodies, respectively. (C) The corresponding magnifiedview of a single cell in (B). (D) The corresponding fluorescence intensity distributions taken from the white lines in (C).Scale bar: 40μm.

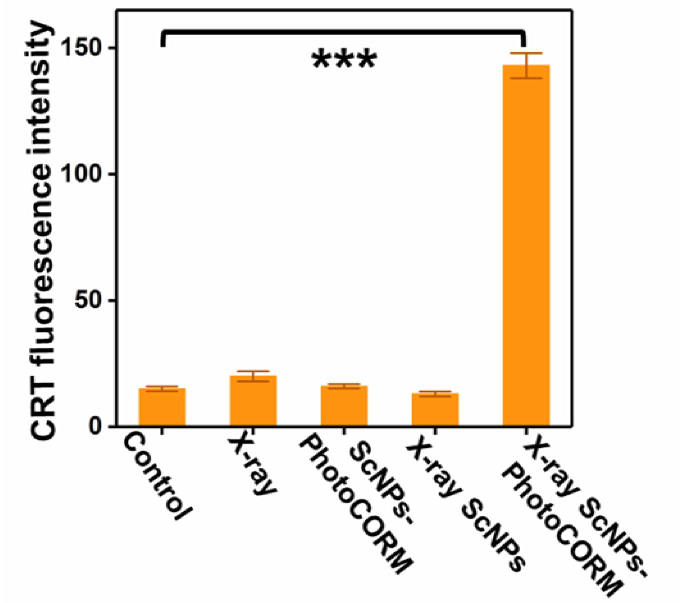

Figure S15. Statistical data of the CRT-positive fluorescence intensity acquired from **Figure S14A**.

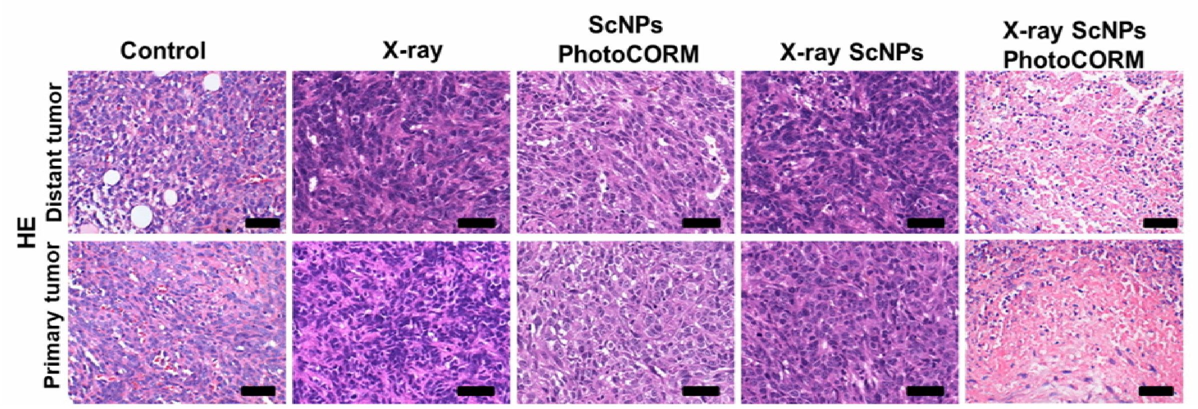

Figure S16.HE stained images of primary and distant tumor slices collected from different test groups. Scale bar: 50µm.

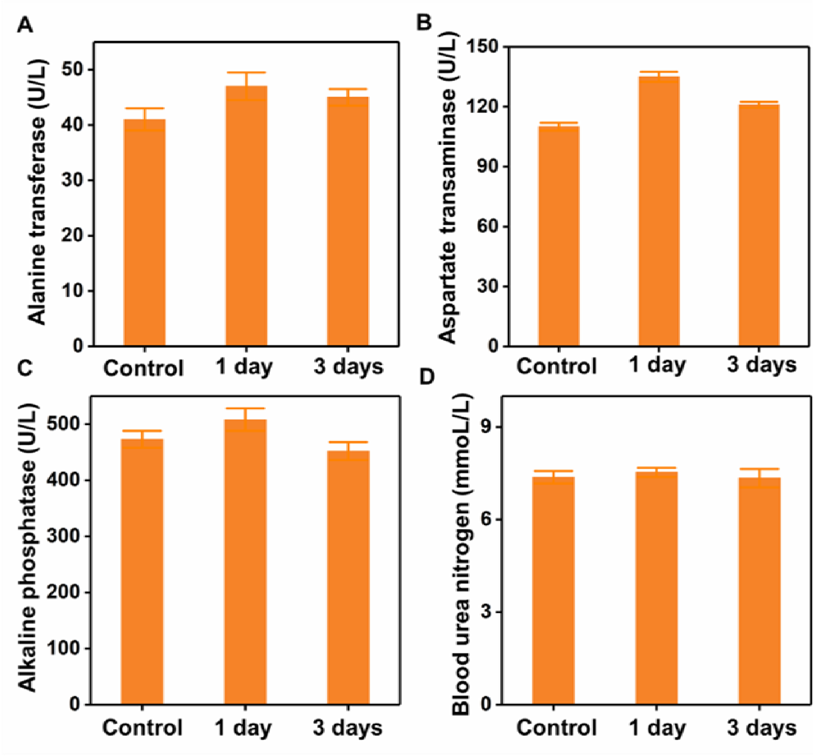

Figure S17.*In vivo*blood biochemistry testsof the test mice after intravenous injection of ScNPs-PhotoCORM nanovaccine and control mice withinjection of PBS.
